# Efficacy of high-dose steroids versus low-dose steroids in the treatment of immune checkpoint inhibitor-associated myocarditis: a case series and systematic review

**DOI:** 10.3389/fimmu.2025.1455347

**Published:** 2025-02-12

**Authors:** Xiuyue Man, Hong Wang, Chen Chen, Xiaofeng Cong, Lemeng Sun, Xueru Sun, Chen Chen, Jing Zhang, Lei Yang

**Affiliations:** Cancer Center, The First Hospital of Jilin University, Changchun, Jilin, China

**Keywords:** immune checkpoint inhibitor-associated myocarditis, ICI-M, corticosteroids, steroids, treatment

## Abstract

**Background:**

Immune checkpoint inhibitor-associated myocarditis (ICI-M) is a rare yet potentially fatal complication of immunotherapy, with no standardized treatment protocol due to limited data. The use of varying steroid doses has resulted in inconsistent outcomes.

**Methods:**

We retrospectively identified patients diagnosed with ICI-M at our institution between January 2020 and February 2024. Additionally, we conducted a comprehensive literature review using PubMed, Embase, and the Cochrane Library to facilitate a comparative analysis of clinical responses. The primary aim was to compare clinical outcomes and therapeutic responses between patients treated with high-dose versus low-dose methylprednisolone.

**Results:**

Patients receiving an initial high-dose intravenous methylprednisolone (1 g/day) exhibited a more rapid reduction in myocardial injury markers, including troponin I/T (cTnI/T), creatine kinase (CK), and N-terminal pro b-type natriuretic peptide (NT-proBNP), compared to those receiving lower doses. This group also demonstrated lower incidences of biomarker rebound and maintained lower levels over time. Additionally, the clinical treatment process was more straightforward in the high-dose group, with treatment efficacy surpassing that observed in patients who received an initial methylprednisolone (mPSL) dose of less than 1 g/day. Regarding prognosis, the incidence of major adverse cardiovascular events (MACE) and cardiovascular mortality was significantly lower in the high-dose group compared to the low-dose group.

**Conclusions:**

In patients with immune checkpoint inhibitor-associated myocarditis, the prompt administration of high-dose corticosteroid pulse therapy (1 g/day) is strongly associated with improved clinical outcomes. This intervention rapidly lowers myocardial injury biomarkers (cTnI/T, CK, NT-proBNP) while minimizing the risk of biomarker rebound, thus optimizing clinical management. Notably, it significantly reduces the incidence of major adverse cardiovascular events (MACE), thereby enhancing patient prognosis. The duration of therapy should be tailored based on clinical response. In cases of steroid resistance, combination therapies may provide additional benefit.

## 1 Introduction

Immune checkpoint inhibitors (ICIs) have revolutionized cancer treatment, providing new hope for patients with various malignancies. However, ICI therapy can induce a range of immune-related adverse events (irAEs), including infusion reactions and off-target effects. Among these, ICI-M is a rare but potentially life-threatening irAE (1). ICI-M can present with diverse clinical features such as myocarditis, pericarditis, arrhythmias, ventricular dysfunction, vasculitis, and endocarditis (2–5). While the estimated incidence of ICI-M is 1%-2%, the true rate may be underreported (6), and its mortality ranges from 25%-50%, making it one of the deadliest irAEs (7).

Currently, no unified treatment strategy for ICI-M has been established by professional societies or consensus guidelines. This lack of consensus is primarily due to the limited availability of systematic data on corticosteroid therapy for ICI-M and the variability in patients' general conditions, underlying diagnoses, and ICI usage (8–14).

In this report, we present a case series of five patients with ICI-M treated at our institution. We describe the changes in clinical symptoms, laboratory findings, and prognosis following initial high-dose corticosteroid therapy. Additionally, a retrospective literature review was conducted to compare treatment responses in ICI-M across different corticosteroid dosages.

## 2 Methods

### 2.1 Institutional case series

#### 2.1.1 Patient selection

A retrospective review was conducted involving patients with immune checkpoint inhibitor (ICI)-related myositis who received treatment at the First Hospital of Jilin University from January 2020 to February 2024.

#### 2.1.2 Diagnostic criteria

According to the consensus statement from the International Cardio-Oncology Society (IC-OS) (15), the diagnosis of this condition is established when there is an elevation in troponin (either new or a significant change from baseline) accompanied by one major criterion, or when there is an elevation in troponin (new or a significant change from baseline) accompanied by two minor criteria, after excluding acute coronary syndrome or acute infectious myocarditis (**Table 1**).

**Table 1:** IC-OS 2021 consensus (15).

Category	Criteria
Diagnostic Requiremen	Either of the following must be present:
	1. Pathohistological diagnosis: Multifocal inflammatory cell infiltrates with overt cardiomyocyte loss by light microscopy of cardiac tissue samples
	2. Clinical diagnosis: A troponin elevation (new or significant change from baseline) plus either a) one major criterion or b) two minor criteria, after excluding acute coronary syndrome or acute infectious myocarditis based on clinical suspicion
Major Criterion	• CMR diagnostic for acute myocarditis (modified Lake Louise criteria)
Minor Criteria	• Clinical syndrome (including any one of the following: fatigue, muscle weakness, myalgias, chest pain, diplopia, ptosis, shortness of breath, orthopnea, lower extremity edema, palpitations, lightheadedness/dizziness, syncope, cardiogenic shock)
	• Ventricular arrhythmia and/or new conduction system disease
	• Decline in cardiac (systolic) function, with or without regional WMA in a non-Takotsubo pattern
	• Other immune-related adverse events, particularly myositis, myopathy, myasthenia gravis
	• Suggestive CMR (meeting some but not all of the modified Lake Louise criteria)

1. Both troponin I and troponin T can be used; however, troponin T may be falsely elevated in those with concomitant myositis. 2. Clinical diagnoses should be confirmed with CMR or endomyocardial biopsy if possible and without causing delays in treatment. 3. In clinically unwell patients, treatment with immunosuppression should be promptly initiated while awaiting further confirmatory testing.

#### 2.1.3 Treatment protocol

High-dose corticosteroid pulse therapy is widely regarded as the first-line treatment for ICI-M. Consequently, for patients diagnosed with ICI-M according to established criteria, high-dose intravenous methylprednisolone (1 g/day) pulse therapy has been incorporated into our standardized treatment protocol.

#### 2.1.4 Data collection

Data extracted from medical records included patient demographics, clinical presentations, treatment patterns, laboratory tests, outcomes, and follow-up information regarding vital and disease status. This follow-up specifically encompassed clinical symptoms, electrocardiography (ECG), ejection fraction (EF), and MACE, which include cardiovascular mortality, myocardial infarction, stroke, heart failure, and cardiac arrest, assessed at three months post-treatment completion.

#### 2.1.5 Ethical considerations

This case series adheres to the CARE guidelines (16). This study received approval from our institutional review board and was granted a waiver for written informed consent (Ethics Approval No.: 2024-671, **Supplementary Figure 6**). All procedures involving human participants in this study were conducted in accordance with the Helsinki Declaration (17).

### 2.2 Systematic review

#### 2.2.1 Search methodology

To identify publications reporting treatment responses in ICI-M, a literature search was conducted in accordance with the Preferred Reporting Items for Systematic Reviews and Meta-Analyses (PRISMA) guidelines for systematic reviews and meta-analyses (18). The systematic search included the PubMed, Embase, and Cochrane Library databases. Controlled vocabulary (MeSH terms) and free-text terms were utilized, including "Immune Checkpoint Inhibitors" and "Myocarditis," which were combined using the Boolean operators "AND" and "OR." Detailed search terms and strings are provided in the **Supplementary Materials** (**Supplementary Table 1**).

#### 2.2.2 Selection criteria

Case reports, case series, or reviews of relevant cases were considered for inclusion. Inclusion criteria were as follows: (1) clinical or pathological diagnosis of ICI-M; (2) initiation of corticosteroid therapy as the primary treatment regimen for ICI-M, with explicit indication of treatment dosage; and (3) availability of laboratory results for cTnI/T, CK, or NT-proBNP. The exclusion criteria included: (1) initial treatment regimens for ICI-M that involved other immunosuppressants, such as Antithymocyte Globulin (ATG), Mycophenolate Mofetil (MMF), infliximab (IFX), Abatacept, Intravenous Immunoglobulin (IVIG), Tocilizumab, or Tofacitinib; and (2) re-administration of immune checkpoint inhibitors following a confirmed diagnosis of ICI-M. Each article was independently reviewed by two evaluators, and any discrepancies were resolved through discussion. If consensus could not be reached, a third reviewer was consulted to minimize bias.

### 2.3 Study endpoints

The objective of this study was to compare the effects of high-dose versus low-dose mPSL therapy on the clinical presentation and therapeutic response in patients with ICI-M. The primary endpoint was the incidence of MACE following the completion of treatment. Secondary endpoints included: (1) the time required to achieve a ≥90% reduction in myocardial injury markers, including cTnI/T, CK, and NT-proBNP; (2) usage patterns of mPSL, including the proportion of re-administration or dose escalation; and (3) changes in cTnI/T levels, specifically the proportion of cases with re-elevation or persistently elevated cTnI/T levels after an initial decline.

### 2.4 Statistical analysis

Continuous data were expressed as median (interquartile range, IQR) or mean ± standard deviation (SD), while categorical data were presented as frequencies (percentages). Differences between continuous variables were compared using t-tests or non-parametric tests, and differences between categorical variables were assessed using the chi-square test (χ²) or Fisher’s exact test. Statistical analysis was performed using SPSS version 27.0. All tests were two-tailed, with a p-value of <0.05 considered indicative of statistical significance.

## 3 Results

### 3.1 Institutional case series

We conducted a comprehensive search for all oncology patients who received immune checkpoint inhibitors at our institution from January 2020 to February 2024. For patients presenting with symptoms suggestive of myocarditis, immediate bedside electrocardiography and echocardiography were performed to assess cardiac function and exclude other potential etiologies. Additionally, biomarkers indicative of myocardial injury were measured to assist in confirming the diagnosis of myocarditis. A diagnosis of ICI-M was established only when the symptoms could not be attributed to alternative diagnoses. These imaging studies and biomarker assessments are integral to our institution’s standard protocol, ensuring that patients receive timely and accurate evaluations to optimize treatment strategies.

In our review, we identified five patients with ICI-M, comprising three females and two males, with a mean age of 66.6 years (range: 65-68 years). Among these patients, three were diagnosed with urologic tumors and two with lung cancer. Notably, two patients (cases 1 and 4) had a history of coronary artery disease.

Prior to diagnosis, all five patients presented with clinical symptoms such as dyspnea and fatigue and underwent electrocardiography and echocardiography evaluations. Upon diagnosis, cardiac injury markers—myoglobin (Mb), creatine kinase MB (CKMB), and cardiac troponin I (cTnI)—were elevated in four patients (cases 1, 2, 3, and 4), while case 5 exhibited elevated cTnI levels only. NT-proBNP levels were elevated in four patients (cases 1, 2, 4, and 5), and myocardial enzymes—creatine kinase (CK), creatine kinase isoenzyme, lactate dehydrogenase (LDH), and α-hydroxybutyrate dehydrogenase (α-HBDH)—were elevated in three patients (cases 1, 2, and 3). Additionally, two patients (cases 2 and 3) were diagnosed with ICI-M in conjunction with other related irAEs, such as myositis (**Table 2**).

**Table 2:** Demographic and patient information of cases at our hospital.

	Case 1	Case 2	Case 3	Case 4	Case 5
Patient Demographics					
Age	65	67	68	68	65
Gender	Male	Female	Female	Male	Female
Tumor Type	Urothelial carcinoma	Non-small cell lung cancer	Urothelial carcinoma	Renal cell carcinoma	Non-small cell lung cancer
Medical History	Hypertension, Diabetes mellitus, Coronary artery disease	Hypertension	Hypertension, Diabetes mellitus	Hypertension, Diabetes mellitus, Coronary artery disease	Absent
ICI Treatment Details					
ICI	Toripalimab	Sintilimab	Toripalimab	Toripalimab	Pembrolizumab
Time from initiation of ICI treatment to diagnosis of ICI-M (days)	31	21	25	22	600
Concomitant with other immune-related adverse events (irAEs)	Absent	Myositis, Rhabdomyolysis, MG, Liver injury	Myositis	Absent	Absent
Immunosuppressive Therapy (IST)	Absent	Absent	IFX, MMF, IVIG	Absent	IVIG
Clinical Presentation and Management					
Clinical Symptoms	Chest discomfort, Weakness, Palpitations	Dyspnea, Chest discomfort, Myalgia, Weakness, Ptosis	Ptosis, Weakness, Myalgia	Dyspnea, Myalgia, Weakness	Palpitations, Dyspnea
Electrocardiogram (ECG)	Sinus tachycardia, ST elevation	ST elevation, Right bundle branch block	Sinus tachycardia、ST elevation	ST elevation	Sinus tachycardia, ST elevation
Pre-treatment values of cardiac injury markers^*^ before mPSL therapy	3682.0ng/ml; 74.20ng/ml; 0.750ng/ml; Within normal limits;	6812.0ng/ml; 194ng/ml; 10.500ng/ml; 3050.0pg/ml	5069.0ng/ml; 104.00ng/ml; 0.949ng/ml; Within normal limits;	2705.0ng/ml; 64.30ng/ml; 15ng/ml; 2700.0pg/ml	Absent; Absent; 6.37ng/ml; 6590.0pg/ml
Pre-treatment values of myocardial enzymes^#^ before mPSL therapy	7420U/L; 143.1U/L; 741U/L; 532U/L;	11601U/L; 339.9U/L; 2171U/L; 1699U/L;	5346U/L; 195.4U/L; 907U/L; 728U/L;	Absent; Absent; 386U/L; Absent;	Absent; Absent; Absent; Absent;
Outcomes and Follow-up					
Clinical Symptoms at 3 Months Post-Treatment	Absent	Absent	Absent	Absent	Absent
Electrocardiogram (ECG) at 3 Months Post-Treatment	Normal	Normal	Normal	Normal	Normal
Ejection Fraction (EF) at 3 Months Post-Treatment	58%	65%	67%	64%	50%
MACE at 3 Months Post-Treatment	No	No	No	No	No
Outcome at 3 Months Post-Treatment	Survival	Disease progression leading to mortality	Survival	Survival	Survival
Number of days with initial ≥90% reduction in cTnI levels after mPSL therapy (days)	10	6	5	6	23
Number of days with initial ≥90% reduction in CK levels after mPSL therapy (days)	4	5	4	5	Absent
Number of days with initial ≥90% reduction in NT-proBNP levels after mPSL therapy (days)	Within normal limits	>35	Within normal limits	>20	>13
Number of days with initial cTnI ≤ULN after mPSL therapy (days)	65	>49	8	>20	>24
Number of days with initial cTnI ≤ULN after mPSL therapy (days)	65	>49	8	>20	>24
Number of days with initial CK ≤ULN after mPSL therapy (days)	5	16	4	5	Absent
Number of days with initial NT-proBNP ≤ULN after mPSL therapy (days)	Within normal limits	>35	Within normal limits	>20	>13

**Table 2** presents detailed case data of five patients diagnosed with ICI-M at our institution. Variables include demographic information, tumor type, immune checkpoint inhibitor (ICI) use, clinical presentation, therapeutic interventions, cardiac injury marker responses to methylprednisolone (mPSL) therapy, and outcomes at 3-month follow-up.

*Cardiac Injury Markers: Myoglobin (Mb, reference range 0-121 ng/ml), Creatine Kinase MB (CK-MB, reference range 0-3.38 ng/ml), cardiac Troponin I (cTnI, reference range 0-0.034 ng/ml), N-terminal pro B-type natriuretic peptide (NT-proBNP, reference range 0-125 pg/ml)

#Myocardial Enzymes: Creatine Kinase (CK, reference range 50-310 U/L), Creatine Kinase Isoenzyme (reference range 0.0-25 U/L), Lactate Dehydrogenase (LDH, reference range 120-250 U/L), α-Hydroxybutyrate Dehydrogenase (α-HBDH, reference range 72.0-182.0 U/L).

All patients received immediate treatment following diagnosis. Each of the five patients was initially administered intravenous high-dose mPSL at a dosage of 1 g/day, which was subsequently tapered based on clinical symptoms and laboratory results. Additionally, two patients received adjunctive therapies: Case 3 was treated with a combination of MMF, IFX, and IVIG, while Case 5 received IVIG as adjunctive treatment. Following the initiation of corticosteroid therapy, four patients (cases 1, 2, 4, and 5) exhibited a downward trend in cTnI and CK levels. During treatment with 1 g/day of mPSL, all patients experienced substantial decreases in both cTnI and CK levels without any rebound phenomenon. However, after tapering the corticosteroids, one patient (case 3) demonstrated a significant increase in cTnI levels after initially normalizing. Electrocardiograms revealed sinus rhythm without any abnormal clinical symptoms or signs. The traditional definition of corticosteroid resistance in ICI-related myocarditis was not applicable in this context. Nevertheless, to manage the elevated cTnI levels, immunosuppressive agents were gradually introduced during the rising phase, accompanied by corresponding increases in the corticosteroid dosage. Throughout the treatment course, two patients (cases 1 and 2) reported no significant discomfort. In contrast, two patients (cases 3 and 4), who had pre-existing diabetes mellitus, experienced glycemic instability, and one patient (case 5) developed a pulmonary fungal infection. Ultimately, all patients were discharged in generally good condition following comprehensive treatment. Unfortunately, none of the five patients resumed anti-tumor therapy and opted for regular follow-up examinations instead.

At the three-month follow-up after the completion of treatment, all patients exhibited resolution of clinical symptoms, with no electrocardiographic abnormalities or occurrences of MACE. Four patients (cases 1, 3, 4, and 5) remained alive with no evidence of disease progression, while one patient (case 2) ultimately succumbed to disease progression (**Supplementary Figures 1-5**).

### 3.2 Systematic review

**Figure 1:**
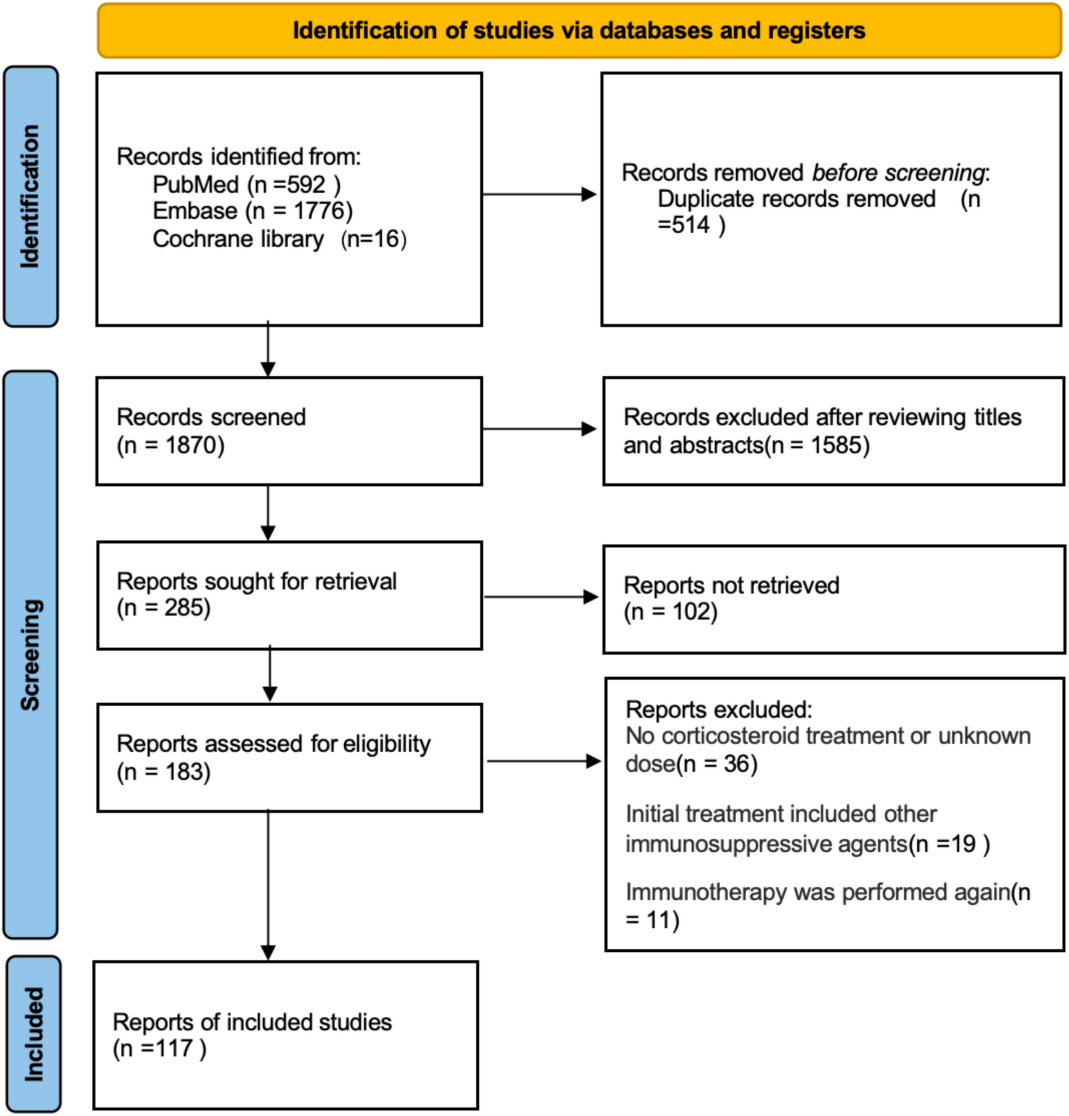
Selection process of research reports using Preferred Reporting Items for Systematic Reviews and Meta-Analyses (PRISMA) for systematic review and meta-analysis.

After reviewing 2,384 articles from PubMed, Embase, and the Cochrane Library, we identified 117 articles involving a total of 141 patients that met our review criteria (**Figure 1**; **Supplementary Table 1**). Among these, 43 patients received an initial intravenous administration of methylprednisolone at a dose of 1 g/day (designated as the high-dose group), while the remaining 98 patients received initial intravenous administration of mPSL at doses less than 1 g/day (designated as the low-dose group) (**Supplementary Tables 2, 3**).

We combined the cases from our hospital with those obtained from a systematic search to analyze the differences in treatment outcomes between the high-dose and low-dose groups. **Table 3** presents the demographic and clinical characteristics of the patients included in the study. The median age of patients with ICI-related myocarditis (n=146) was 67 years (interquartile range [IQR], 60–73), with 58.90% being male. The most common tumor type among these patients was melanoma (25.34%, 37/146), followed by non-small cell lung cancer (NSCLC) (16.44%, 24/146). ICI-related myotoxicity often manifests as concurrent myocarditis (heart involvement) and myositis (skeletal muscle involvement), which is associated with a high mortality rate (19). In addition to myositis, the co-occurrence rates of myasthenia gravis (MG), liver injury, and kidney injury are also notably high (20). Among the 146 patients, a total of 89 experienced two or more irAEs concurrently.

**Table 3:** Comparison of demographic and clinical characteristics between high-dose and low-dose groups.

		High-Dose Groups (n=48)	Low-Dose Groups (n=98)
Baseline Demographic Characteristics			
Median age at onset of ICI-M (years) [M (P25, P75)]		67 (60, 73)	
Median time to onset of ICI-M (days) [M (P25, P75)]		28 (19.25, 45.75)	
Gender (n, %)	Female	15, 31.25%	45, 45.92%
	Male	33, 68.75%	53, 54.08%
Country (n, %)	China	12, 25.00%	31, 31.63%
	Excluding China	36, 75.00%	67, 68.36%
Clinical Features at Presentation			
Tumor type (n, %)	Melanoma	11, 22.92%	26, 26.53%
	Non-small cell lung cancer	10, 20.83%	14, 14.29%
	Thymoma	4, 8.33%	7, 7.14%
	Other types	23, 47.92%	51, 52.04%
Medical history (n, %)	Exist	21, 43.75%	43, 43.88%
	Absent	27, 56.25%	55, 56.12%
ICI (n, %)	PD-1	30, 62.50%	70, 71.43%
	PD-L1	4, 8.33%	4, 4.08%
	CTLA-4	0, -	1, 1.02%
	Combination therapy	14, 29.17%	23, 23.47%
Clinical symptoms (n, %)	Exist	43, 89.58%	86, 87.76%
	Absent	5, 10.42%	12, 12.24%
Electrocardiogram (ECG) (n, %)	Abnormality	33, 68.75%	65, 66.33%
	Normality	6, 12.50%	15, 15.31%
	Unknown	9, 18.75%	18, 18.37%
Ejection Fraction (EF) (n, %)	<45%	11, 22.92%	15, 15.31%
	≥45%	22, 45.83%	57, 58.16%
	Unknown	15, 31.25%	26, 26.53%
Other irAEs (n, %)	Exist	26, 54.17%	63, 64.29%
	Absent	22, 45.83%	35, 35.71%
Therapeutic Interventions and Outcomes			
MACE after Treatment Completion (n, %)	Cardiovascular death	3, 6.25%	23, 23.47%
	Myocardial Infarction	0, -	1, 1.02%
	Stroke	0, -	1, 1.02%
	Heart failure	1, 2.08%	4, 4.08%
	Cardiac arrest	0, -	1, 1.02%
	No	44, 91.67%	68, 69.39%

**Table 3** summarizes the baseline demographic data, clinical features at presentation, therapeutic interventions, and outcomes of patients treated with high-dose and low-dose corticosteroids for ICI-M. All the aforementioned statistics were conducted within the subset of patients with clearly available relevant data, with unknown patients excluded.

Notably, ICI-related myocarditis occurred predominantly after PD-1 treatment in 68.49% of cases. Due to insufficient data on the number of patients who did not develop ICI-related myocarditis following either monotherapy or combination therapy, we could not determine the incidence rates associated with different treatment regimens. However, existing studies suggest that the mortality associated with combination therapy involving anti-PD-1/PD-L1 and anti-CTLA-4 is significantly higher than that observed with anti-PD-1/PD-L1 monotherapy (21, 22). Additionally, the incidence rate of immune checkpoint inhibitor combination therapy is significantly greater than that of monotherapy (23). The median time to the onset of ICI-related myocarditis after immune checkpoint inhibitor treatment was 28 days (interquartile range [IQR], 19.25–49.75).

Previous studies have indicated that approximately 90% of patients with ICI-M present with non-specific clinical symptoms at the initial stage, such as ptosis and fatigue (24). Furthermore, around 90% of patients exhibit varying degrees of electrocardiographic abnormalities, although only a small proportion experience a decrease in EF (25). In our dataset, 88.36% (129/146) of patients displayed clinically relevant symptoms. When ICI-M was suspected, the majority of patients presented with electrocardiographic abnormalities (82.35%, 98/121), while a smaller percentage had an EF <45% (24.76%, 26/105).

Compared to the low-dose group, the proportion of patients in the high-dose group who experienced an increase or sustained elevation in cTnI/T after an initial decline during treatment was significantly lower (38.64%, 17/44 vs. 69.14%, 56/81; p=0.001). Fewer patients in the high-dose group required re-administration or dosage escalation of mPSL, indicating a significant difference (4.17%, 2/48 vs. 41.67%, 30/96; p=0.000). Additionally, in the high-dose group, the proportion of patients requiring subsequent initiation of immunosuppressive therapy (IST) during treatment was significantly lower compared to the low-dose group (58.33%, 28/48 vs. 64.29%, 63/98; p=0.026). Although the overall mortality rate was higher in the low-dose group, this difference was not statistically significant (30.43%, 14/46 vs. 46.24%, 43/93; p=0.075). This lack of significance may be attributed to the limited sample size, which reduced the statistical power of the analysis. Nonetheless, the observed trend may carry clinical relevance, suggesting the potential importance of dose in influencing outcomes. However, cardiovascular mortality was significantly lower in the high-dose group (21.43%, 3/14 vs. 53.49%, 23/43; p=0.036). Furthermore, the incidence of MACE following treatment completion was significantly lower in the high-dose group (8.33%, 4/48 vs. 30.61%, 30/98; p=0.003), suggesting a better prognosis (**Table 4**). To ensure the accuracy of the results, patients classified as 'unknown' in the outcome categories were excluded to mitigate the influence of uncertain data on the findings.

**Table 4:** Comparative analysis between high and low dosage groups.

		High-Dose Groups (n=48)	Low-Dose Groups (n=98)	Test Statistic	P-value
Efficacy of mPSL Therapy and Follow-Up Results					
Elevation or sustained elevation of cTnI/T (n, %)	Yes	17, 38.64%	56, 69.14%	10.917^*^	0.001
	No	27, 61.36%	25, 30.86%		
	Unknown	4, -^¥^	17, -		
Reinitiation of mPSL or escalation of mPSL dosage (n, %)	Yes	2, 4.17%	40, 41.67%	21.742^*^	0.000
	No	46, 95.83%	56, 58.33%		
	Unknown	0, -	2, -		
Reinitiation of IST (n, %)	Yes	28, 58.33%	63, 64.29%	4.971^*^	0.026
	No	20, 41.67%	35, 35.71%		
Post-Treatment Outcomes and Complications					
MACE after Treatment Completion (n, %)	Yes	4, 8.33%	30, 30.61%	8.952^*^	0.003
	No	44, 91.67%	68, 69.39%		
Prognosis (n, %)	Mortality	14, 30.43%	43, 46.24%	3.177^*^	0.075
	Survival	32, 88.89%	50, 53.76%		
	Unknown	2, -	5, -		
Mortality (n, %)	Cardiovascular death	3, 21.43%	23, 53.49%	4.376^*^	0.036
	Non-cardiovascular death	11, 78.57%	20, 46.51%		
Biomarker-Based Prognostic Stratification					
Prognosis with cTnI/T ≥ 32 ULN (n, %)	Mortality	11, 36.67%	31, 56.36%	3.013^*^	0.083
	Survival	19, 63.33%	24, 43.64%		
	Unknown	3, -	3, -		
Prognosis with cTnI/T < 32 ULN (n, %)	Mortality	2, 16.67%	9, 30.00%	0.000^†^	0.464
	Survival	10, 83.33%	21, 70.00%		
	Unknown	0, -	2, -		

**Table 4** provides a comparative analysis of clinical outcomes in patients receiving high-dose versus low-dose glucocorticoid therapy. It highlights key prognostic factors, including changes in cardiac biomarkers (cTnI/T), adjustments in treatment regimens, incidence of major adverse cardiac events (MACE), and mortality rates. Subgroup analyses based on cTnI/T levels further explore variations in prognosis to elucidate the potential differential impacts of treatment doses.

*Chi-square Test.

^†^Fisher’s Exact Test.

^¥^The analysis of differences excluded patients with an outcome category of "unknown," ensuring the accuracy of the results and avoiding the impact of uncertain data.

Lehmann et al. suggested that a peak cTnT/ULN ≥ 32 indicates a high-risk group, which correlates with an increased overall mortality rate and a higher risk of MACE (26). Consequently, we combined patients from both the high-dose and low-dose groups, hypothesizing that a similar poor prognosis would be observed for patients with peak cTnI or cTnT/ULN ≥ 32. In a cohort of 135 patients with pre-treatment peak cTnI or cTnT measurements, we stratified them into high-risk (peak cTnI or cTnT/ULN ≥ 32) and low-risk groups (peak cTnI or cTnT/ULN < 32). Among the high-risk group, 33 patients received initial high-dose corticosteroid treatment, while 58 received low-dose corticosteroids. The mortality rate was lower in the high-dose group (36.67%, 11/30 vs. 56.36%, 31/55; p=0.083). In the low-risk group, 12 patients received initial high-dose corticosteroid treatment, and 32 patients received low-dose corticosteroids, with the high-dose group also demonstrating a lower mortality rate (16.67%, 2/12 vs. 30.00%, 9/30; p=0.464) (**Table 4**).

In addition, we performed a more detailed stratification of the initial glucocorticoid doses within the low-dose group and subsequently calculated the post-treatment incidence of MACE and mortality rates, excluding 5 patients with unknown prognoses (**Table 5**). Among patients receiving doses of ≤1 mg/kg/d (n = 34), the incidence of MACE was 38.24% (13/34), and the mortality rate was 48.39% (15/31). In the >1 mg/kg/d group (n = 23), the MACE incidence was 34.78% (8/23), and the mortality rate was 63.64% (14/22). Furthermore, considering that some patients received fixed doses, we analyzed two subgroups separately: For the 5 ~ 480 mg/d subgroup (n = 31), the incidence of MACE was 25.81% (8/31), and the mortality rate was 40.00% (12/30). In the 500 mg/d subgroup (n = 10), the MACE incidence was 10.00% (1/10), and the mortality rate was 20.00% (2/10).

**Table 5:** Dosage distribution and corresponding MACE and mortality rates in the low-dose group.

Dosage (mg/kg/d or mg/d)	Total Patients (n, %)	Patients with Post-Treatment MACE (n, %)	Mortality (n, %)
**≤1 mg/kg/d**	**34, 34.69%**	**13, 38.24%**	15, 48.39%^*^
0.2 mg/kg/d	1, 1.02%	1, 100.00%	0, -
0.5 mg/kg/d	3, 3.06%	1, 33.33%	1, 33.33%
1 mg/kg/d	30, 30.61%	11, 40.74%	14, 51.85%^*^
**>1mg/kg/d**	**23, 23.47%**	**8, 34.78%**	14, 63.64%^*^
1.5 mg/kg/d	2, 2.04%	0, -	2, 100.00%
2 mg/kg/d	20, 20.41%	8, 40.00%	12, 63.16%^*^
4 mg/kg/d	1, 1.02%	0, -	0, -
**5-480 mg/d**	**31, 31.63%**	**8, 25.81%**	12, 40.00%^*^
5 mg/d	1, 1.02%	0, -	0, -^*^
15 mg/d	1, 1.02%	0, -	0, -
16 mg/d	1, 1.02%	1, 100.00%	0, -
20 mg/d	1, 1.02%	0, -	0, -
30 mg/d	1, 1.02%	0, -	1, 100.00%
40 mg/d	3, 3.06%	1, 33.33%	1, 33.33%
80 mg/d	4, 4.08%	1, 25.00%	1, 25.00%
120 mg/d	4, 4.08%	2, 50.00%	2, 50.00%
125 mg/d	3, 3.06%	0, -	0, -
200 mg/d	4, 4.08%	1, 25.00%	2, 50.00%
240 mg/d	2, 2.04%	0, -	1, 50.00%
250 mg/d	1, 1.02%	0, -	1, 100.00%
320 mg/d	2, 2.04%	1, 50.00%	1, 50.00%
360 mg/d	1, 1.02%	1, 100.00%	1, 100.00%
480 mg/d	2, 2.04%	0, -	1, 50.00%
**500 mg/d**	**10, 10.20%**	**1, 10.00%**	**2, 20.00%**
**Total**	**98, 100%**	**30, 30.61%**	43, 46.24%^*^

**Table 5** summarizes the dosage distribution of corticosteroids in the low-dose group and evaluates its association with post-treatment MACE and mortality rates. Dosage is reported in mg/kg/day or mg/day.

^*^Five patients with unknown prognosis were excluded from mortality rate calculations (1 mg/kg/d: 3 patients; 5 mg/d: 1 patient; 2 mg/kg/d: 1 patient).

The bold values represent the main dosage categories.

Among the 146 cases, we identified substantial changes in cTnI/T, CK, and NT-proBNP levels during the treatment process, with 17 cases in the high-dose group and 23 cases in the low-dose group. The results demonstrated that the median time to achieve an initial reduction of cTnT levels by ≥90% following mPSL treatment was significantly shorter in the high-dose group compared to the low-dose group (15.0 days vs. 44.5 days, P = 0.007). However, no significant differences were observed between the two groups regarding the median time to achieve a ≥90% reduction in cTnI (8.0 days vs. 11.5 days, P = 0.335), CK (7.5 days vs. 7.0 days, P = 0.859), or NT-proBNP levels (21.0 days vs. 8.0 days, P = 0.468). To ensure the accuracy of the results, patients without continuous monitoring were excluded from the analysis (**Table 6**; **Supplementary Tables 4, 5**).

**Table 6:** Time to ≥90% decline in cTnI/T, CK, and NT-proBNP levels in high and low dosage groups.

	High-Dose Groups		Low-Dose Groups		Z-value	P-value
	N	Median time (days) [M (P25, P75)]	N	Median time (days) [M (P25, P75)]		
Initial cTnI reduction ≥90% after mPSL treatment	9	8.0 (6, 15.5)	12	11.5 (7, 16.75)	-0.965	0.335
Initial cTnT reduction ≥90% after mPSL treatment	11	15.0 (5, 29)	10	44.5 (23.75, 65.25)	-2.681	0.007
Initial CK reduction ≥90% after mPSL treatment	13	7.5 (5.25, 20.75)	17	7.0 (5, 13.5)	-0.178	0.859
Initial NT-proBNP reduction ≥90% after mPSL treatment	1	21.0 (21.0, 21.0)	4	8.0 (1, 48.75)	-0.725	0.468

**Table 6** presents the time required for patients treated with high-dose and low-dose methylprednisolone (mPSL) to achieve a ≥90% decline in cTnI/T, CK, and NT-proBNP levels. This analysis compares the distribution of recovery times between the two dosage groups, aiming to evaluate the effectiveness of different dosage regimens in reducing key cardiac injury markers. Patients who did not undergo continuous monitoring were excluded to ensure the accuracy of the results.

## 4 Discussion

Currently, there is no standardized treatment for ICI-M, and the literature on the comparative efficacy and prognosis of high-dose (1 g/day) versus low-dose (<1 g/day) corticosteroid therapy is limited. This study aims to analyze these differences and establish that timely diagnosis and administration of high-dose intravenous methylprednisolone (1 g/day) pulse therapy are superior to low-dose intravenous methylprednisolone (<1 g/day) regarding efficacy and prognosis.

In our hospital case series and literature review, several advantages of high-dose therapy over low-dose therapy were identified. Firstly, high-dose therapy led to more rapid and stable declines in cardiac injury biomarkers (cTnI/T, CK, NT-proBNP) compared to low-dose therapy, regardless of patient risk levels. Secondly, the treatment process was more straightforward in the high-dose group, with patients experiencing a quicker resolution of symptoms. Lastly, the incidence of MACE and cardiovascular mortality following treatment completion was significantly lower in the high-dose group compared to the low-dose group.

### 4.1 Changes in cardiac injury biomarkers

Extensive research has demonstrated that cTnI/T, CK, and NT-proBNP are pivotal biological markers for diagnosing, monitoring changes in, and predicting the prognosis of ICI-M (23, 27). Our results indicate that, compared to the low-dose group, the high-dose group exhibited (1) a lower proportion of patients with persistent elevation or re-elevation of cTnI/T after an initial decline, with rates of 38.64% (17/44) in the high-dose group versus 69.14% (56/81) in the low-dose group. Additionally, the high-dose group demonstrated a greater number of patients with consistently stable declines in cTnI/T levels (**Table 4**). (2) among patients with known approximate changes in cTnI/T, CK, and NT-proBNP, the high-dose group required shorter durations to achieve a ≥90% decline compared to the low-dose group (**Table 6**). Mahmood et al. confirmed that patients receiving higher initial doses of corticosteroids experienced a faster decline in serum troponin levels, ultimately achieving lower levels (28). While most cases identified in our systematic search lacked data on the time to normalization of laboratory values, the observed time required for a ≥90% decline in cTnI/T, CK, and NT-proBNP suggests that the high-dose group may experience shorter normalization times. This underscores the potential efficacy of high-dose corticosteroids in mitigating cardiac injury associated with immune-related adverse events.

### 4.2 Variation in corticosteroid treatment dosage and clinical symptomatology

(1) Compared to the low-dose group (41.67%, 40/96), the proportion of patients in the high-dose group requiring corticosteroid re-initiation or dosage escalation was significantly lower at 4.17% (2/48, p=0.000). This finding highlights a strong association between the dosage of corticosteroids administered and the subsequent need for re-initiation or dosage adjustment (14, 28–31). Moreover, our analysis indicates that the high-dose corticosteroid group demonstrated a significantly lower rate of subsequent initiation of immunosuppressive therapy (IST) compared to the low-dose group (58.33%, 28/48 vs. 64.29%, 63/98; p=0.026). This finding highlights the therapeutic impact of high-dose corticosteroid regimens, potentially reducing the reliance on additional IST for disease management (**Table 4**). (2) Furthermore, a greater number of patients in the high-dose group experienced resolution of initial clinical symptoms related to ICI-M, and the time to normalization of abnormal electrocardiogram values and ejection fraction was shorter. Streamlining the treatment process is essential for achieving successful outcomes, providing significant benefits for patients, their families, and healthcare providers (32). The clinical treatment regimen for patients in the high-dose group is more efficient, resulting in a shorter overall treatment duration compared to the low-dose group. This efficiency translates into lower treatment costs and reduced demands on time and manpower. Additionally, patient emotions significantly influence treatment outcomes. A positive treatment experience can foster confidence in patients, encouraging a more proactive approach to their care, which may lead to improved prognoses.

### 4.3 Regarding prognosis

Based on the data obtained, (1) the incidence of MACE following the completion of treatment was significantly lower in the high-dose group compared to the low-dose group. This finding indicates that high-dose corticosteroid pulse therapy provides superior protection against MACE in patients with ICI-related myocarditis. Additionally, the rate of cardiovascular mortality was significantly lower in the high-dose group, suggesting that high-dose corticosteroid therapy is associated with improved patient outcomes (**Table 4**). Although the overall difference in mortality rates observed in this study did not reach statistical significance (30.43%, 14/46 vs. 46.24%, 43/93, p = 0.075), we propose that this finding warrants further validation in studies with larger sample sizes. Such studies may better elucidate the potential clinical relevance of this trend and provide more definitive evidence to guide therapeutic decision-making. (2) Regardless of risk group, the initiation of high-dose corticosteroid therapy (1 g/day) results in better outcomes, characterized by lower mortality rates and an enhanced prognosis for patients with ICI-related myocarditis.

The findings indicate significant differences in treatment outcomes between the high-dose and low-dose groups. The high-dose group exhibits superior treatment efficacy, enhanced clinical recovery, and improved prognosis. Current guidelines for managing adverse events associated with immune checkpoint inhibitors recommend the timely administration of high-dose corticosteroids for 3 days as the first-line approach. In cases that are refractory to this treatment, additional immunosuppressive agents, such as IFX and MMF, may be considered (10, 11, 13). A retrospective study involving 126 patients undergoing corticosteroid therapy for immune checkpoint inhibitor-related myocarditis supports the benefits of higher initial doses and the early initiation of treatment (33). At our institution, all patients received prompt high-dose corticosteroid pulse therapy upon admission, with most experiencing rapid symptom improvement and favorable outcomes. This underscores the critical importance of early and aggressive corticosteroid therapy in the management of immune checkpoint inhibitor-related myocarditis.

However, corticosteroid therapy can be a double-edged sword, as high-dose corticosteroid treatment for irAEs may reduce overall survival in cancer patients (34–36). A recent study demonstrated that high peak corticosteroid doses were associated with poorer progression-free survival (PFS) and overall survival (OS), while cumulative doses did not show a correlation (37). Eggermont et al. reported that in stage III melanoma patients receiving adjuvant pembrolizumab therapy post-surgery, the use of glucocorticoids (GCC) for more than 30 days negatively impacted recurrence-free survival (RFS) (38). Furthermore, multiple studies have indicated that high-dose corticosteroid treatment for irAEs may have detrimental effects (39, 40). It remains unclear whether these adverse effects stem from specific drugs or from the overall impact of aggressive immunosuppression. Determining whether short-term high-dose corticosteroid therapy or prolonged low-dose therapy is more harmful is crucial for guiding corticosteroid escalation and tapering strategies. This issue warrants further investigation (34, 41).

Our study primarily aims to identify the optimal therapeutic strategy for ICI-M. A comprehensive understanding of its incidence, diagnostic criteria, and subsequent treatment interventions is crucial for effective disease management. The onset of ICI-related myocarditis following treatment with ICIs is influenced by various factors, including patient characteristics, the type and dosage of ICIs, and whether monotherapy or combination therapy is employed. According to the National Comprehensive Cancer Network (NCCN) guidelines, symptoms of ICI-M can manifest within days to weeks after one to two doses of ICIs (13). Escudier et al. reported a median onset time of 65 days for symptom onset (29), with most cases detected within the first three months (28, 42). Our study findings indicate considerable variability in the onset time of ICI-M, ranging from as early as the same day of ICI administration to several years later. The median time to ICI-M onset in our cohort was 28 days (interquartile range [IQR], 19.5–46.5). These findings underscore the importance of immediate and regular monitoring following ICI administration, particularly during the first one to two cycles of treatment, when the risk for myocarditis is heightened.

The ESC guidelines recommend monitoring cardiac troponin (cTn), B-type natriuretic peptide (BNP)/N-terminal pro b-type natriuretic peptide (NT-proBNP), and electrocardiograms (ECG) during the first three cycles of immune checkpoint inhibitor therapy, with echocardiography advised for high-risk cases (12). Vigilance for atypical symptoms is essential, as is the need for prompt evaluations in suspected cases of myocarditis (8, 43). The risk of immune-related adverse events recurrence increases during corticosteroid tapering, underscoring the importance of tailoring therapy duration and tapering regimens based on clinical response and treatment goals (44). While the efficacy of biologics such as antithymocyte globulin (ATG) and Abatacept remains uncertain, personalized combination therapy with other immunosuppressive agents may be necessary, depending on individual patient considerations (45). The mortality risk associated with the re-administration of immunotherapy following the occurrence of immune-related myocarditis is significantly elevated, often leading patients to discontinue further immunotherapy (46). This raises important questions regarding the safety of re-initiating immunotherapy in ICI-M patients, particularly those treated early with high-dose corticosteroid pulse therapy. Further research is needed to explore the selection of appropriate immunotherapy regimens, treatment strategies, and patient prognosis in such cases.

## 5 Conclusion

In summary, myocarditis following immune checkpoint inhibitor treatment may occur more frequently than previously recognized and often manifests early in the treatment course, responding well to higher doses of corticosteroids. However, given that this study is based on a small case series and retrospective analysis, these findings should be interpreted with caution. Early high-dose corticosteroid pulse therapy may offer an effective treatment option for patients with ICI-related myocarditis; nonetheless, further research is essential to validate these results and provide more conclusive evidence.

## 6 Limitation

Our study has several limitations: 1. We excluded articles in languages other than English, which may have resulted in the omission of relevant studies not indexed in SCIE or lacking available full texts, as well as unpublished work. 2. As a retrospective study, continuous clinical data over time were not available. 3. Clinical data for patients in the systematic review were inferred from articles and figures, leading to potential inaccuracies. 4. Both our hospital cases and the systematic review cases lacked consistent, regular time points for key examinations, such as electrocardiograms and echocardiography, which would have provided more detailed insights into treatment outcomes. 5. Missing data in the systematic review may have introduced bias. 6. The limited sample size in this study highlights the need for prospective research involving larger patient cohorts to further substantiate our findings.

## Data Availability

The original contributions presented in the study are included in the article/**Supplementary Material**. Further inquiries can be directed to the corresponding author.

## References

[1] Tajiri K Ieda M . Cardiac complications in immune checkpoint inhibition therapy. Front Cardiovasc Med. (2019) 6:3. doi: 10.3389/fcvm.2019.00003 30729114 PMC6351438

[2] Li C Bhatti SA Ying J . Immune checkpoint inhibitors-associated cardiotoxicity. Cancers (Basel). (2022) 14:1145. doi: 10.3390/cancers14051145 35267453 PMC8909315

[3] Puzanov I Diab A Abdallah K Bingham CO 3rd Brogdon C Dadu R . Managing toxicities associated with immune checkpoint inhibitors: consensus recommendations from the Society for Immunotherapy of Cancer (SITC) Toxicity Management Working Group. J Immunother Cancer. (2017) 5:95. doi: 10.1186/s40425-017-0300-z 29162153 PMC5697162

[4] Johnson DB Balko JM Compton ML Chalkias S Gorham J Xu Y . Fulminant myocarditis with combination immune checkpoint blockade. N Engl J Med. (2016) 375:1749–55. doi: 10.1056/NEJMoa1609214 PMC5247797 27806233

[5] Zito C Manganaro R Ciappina G Spagnolo CC Racanelli V Santarpia M . Cardiotoxicity induced by immune checkpoint inhibitors: what a cardio-oncology team should know and do. Cancers (Basel). (2022) 14:5403. doi: 10.3390/cancers14215403 36358830 PMC9653561

[6] Rubio-Infante N Ramírez-Flores YA Castillo EC Lozano O García-Rivas G Torre-Amione G . Cardiotoxicity associated with immune checkpoint inhibitor therapy: a meta-analysis. Eur J Heart Fail. (2021) 23:1739–47. doi: 10.1002/ejhf.2289 34196077

[7] Awadalla M Mahmood SS Groarke JD Hassan MZO Nohria A Rokicki A . Global longitudinal strain and cardiac events in patients with immune checkpoint inhibitor-related myocarditis. J Am Coll Cardiol. (2020) 75:467–78. doi: 10.1016/j.jacc.2019.11.049 PMC7067226 32029128

[8] Raschi E Rossi S De Giglio A Fusaroli M Burgazzi F Rinaldi R . Cardiovascular toxicity of immune checkpoint inhibitors: A guide for clinicians. Drug Saf. (2023) 46:819–33. doi: 10.1007/s40264-023-01320-5 PMC10442274 37341925

[9] Bermas BL Zaha VG . Mending broken hearts. Circulation. (2021) 143:767–9. doi: 10.1161/CIRCULATIONAHA.120.052307 33617309

[10] Haanen J Obeid M Spain L Carbonnel F Wang Y Robert C . Management of toxicities from immunotherapy: ESMO Clinical Practice Guideline for diagnosis, treatment and follow-up. Ann Oncol. (2022) 33:1217–38. doi: 10.1016/j.annonc.2022.10.001 36270461

[11] Naidoo J Murphy C Atkins MB Brahmer JR Champiat S Feltquate D . Society for Immunotherapy of Cancer (SITC) consensus definitions for immune checkpoint inhibitor-associated immune-related adverse events (irAEs) terminology. J Immunother Cancer. (2023) 11:e006398. doi: 10.1136/jitc-2022-006398 37001909 PMC10069596

[12] Lyon AR López-Fernández T Lancellotti P Thuny F Abdelhamid M Aboyans V . 2022 ESC Guidelines on cardio-oncology developed in collaboration with the European Hematology Association (EHA), the European Society for Therapeutic Radiology and Oncology (ESTRO) and the International Cardio-Oncology Society (IC-OS). Eur Heart J Cardiovasc Imaging. (2022) 23:E333–465. doi: 10.1093/ehjci/jeac106 36017575

[13] Thompson JA Schneider BJ Brahmer J Achufusi A Armand P Berkenstock MK . Management of immunotherapy-related toxicities, version 1.2022, NCCN clinical practice guidelines in oncology. J Natl Compr Cancer Network. (2022) 20:387–405. doi: 10.6004/jnccn.2022.0020 35390769

[14] Brahmer JR Lacchetti C Schneider BJ Atkins MB Brassil KJ Caterino JM . Management of immune-related adverse events in patients treated with immune checkpoint inhibitor therapy: American society of clinical oncology clinical practice guideline. J Clin Oncol. (2018) 36:1714–68. doi: 10.1200/JCO.2017.77.6385 PMC6481621 29442540

[15] Herrmann J Lenihan D Armenian S Barac A Blaes A Cardinale D . Defining cardiovascular toxicities of cancer therapies: an International Cardio-Oncology Society (IC-OS) consensus statement. Eur Heart J. (2022) 43:280–99. doi: 10.1093/eurheartj/ehab674 PMC8803367 34904661

[16] Gagnier JJ Kienle G Altman DG Moher D Sox H Riley D . The CARE guidelines: consensus-based clinical case reporting guideline development. Glob Adv Health Med. (2013) 2:38–43. doi: 10.7453/gahmj.2013.008 PMC3833570 24416692

[17] Rickham PP . Human experimentation. code of ethics of the world medical association. declaration of helsinki. Br Med J. (1964) 2:177. doi: 10.1136/bmj.2.5402.177 14150898 PMC1816102

[18] Zorzela L Loke YK Ioannidis JP Golder S Santaguida P Altman DG . PRISMA harms checklist: improving harms reporting in systematic reviews. BMJ. (2016) 353:i2229. doi: 10.1136/bmj.i2229 26830668

[19] Salem JE Bretagne M Abbar B Leonard-Louis S Ederhy S Redheuil A . Abatacept/ruxolitinib and screening for concomitant respiratory muscle failure to mitigate fatality of immune-checkpoint inhibitor myocarditis. Cancer Discovery. (2023) 13:1100–15. doi: 10.1158/2159-8290.CD-22-1180 36815259

[20] Heinzerling L Ott PA Hodi FS Husain AN Tajmir-Riahi A Tawbi H . Cardiotoxicity associated with CTLA4 and PD1 blocking immunotherapy. J Immunother Cancer. (2016) 4:50. doi: 10.1186/s40425-016-0152-y 27532025 PMC4986340

[21] Moslehi JJ Salem JE Sosman JA Lebrun-Vignes B Johnson DB . Increased reporting of fatal immune checkpoint inhibitor-associated myocarditis. Lancet. (2018) 391:933. doi: 10.1016/S0140-6736(18)30533-6 PMC6668330 29536852

[22] Jo W Won T Daoud A Čiháková D . Immune checkpoint inhibitors associated cardiovascular immune-related adverse events. Front Immunol. (2024) 15:1340373. doi: 10.3389/fimmu.2024.1340373 38375475 PMC10875074

[23] Upadhrasta S Elias H Patel K Zheng L . Managing cardiotoxicity associated with immune checkpoint inhibitors. Chronic Dis Transl Med. (2019) 5:6–14. doi: 10.1016/j.cdtm.2019.02.004 30993259 PMC6450824

[24] Furukawa A Tamura Y Taniguchi H Kawamura A Nagase S Hayashi A . Prospective screening for myocarditis in cancer patients treated with immune checkpoint inhibitors. J Cardiol. (2023) 81:63–7. doi: 10.1016/j.jjcc.2022.07.009 35953399

[25] Pradhan R Nautiyal A Singh S . Diagnosis of immune checkpoint inhibitor-associated myocarditis: A systematic review. Int J Cardiol. (2019) 296:113–21. doi: 10.1016/j.ijcard.2019.07.025 31327516

[26] Lehmann LH Heckmann MB Bailly G Finke D Procureur A Power JR . Cardiomuscular biomarkers in the diagnosis and prognostication of immune checkpoint inhibitor myocarditis. Circulation. (2023) 148:473–86. doi: 10.1161/CIRCULATIONAHA.123.062405 PMC10527069 37317858

[27] Ganatra S Neilan TG . Immune checkpoint inhibitor-associated myocarditis. Oncologist. (2018) 23:879–86. doi: 10.1634/theoncologist.2018-0130 PMC6156176 29802219

[28] Mahmood S Fradley MG Cohen JV Nohria A Reynolds KL Heinzerling LM . Myocarditis in patients treated with immune checkpoint inhibitors. J Am Coll Cardiol. (2018) 71:1755–64. doi: 10.1016/j.jacc.2018.02.037 29567210 PMC6196725

[29] Escudier M Cautela J Malissen N Ancedy Y Orabona M Pinto J . Clinical features, management, and outcomes of immune checkpoint inhibitor-related cardiotoxicity. Circulation. (2017) 136:2085–7. doi: 10.1161/CIRCULATIONAHA.117.030571 29158217

[30] Zhang L Zlotoff DA Awadalla M Mahmood SS Nohria A Hassan MZO . Major adverse cardiovascular events and the timing and dose of corticosteroids in immune checkpoint inhibitor-associated myocarditis. Circulation. (2020) 141:2031–4. doi: 10.1161/CIRCULATIONAHA.119.044703 PMC7301778 32539614

[31] Puzanov I Subramanian P Yatsynovich YV Jacobs DM Chilbert MR Sharma UC . Clinical characteristics, time course, treatment and outcomes of patients with immune checkpoint inhibitor-associated myocarditis. J ImmunoTherapy Cancer. (2021) 9:e002553corr1. doi: 10.1136/jitc-2021-002553 PMC8231054 34162715

[32] Dong S Wang Z Zhang JT Yan B Zhang C Gao X . Circulating tumor DNA-guided de-escalation targeted therapy for advanced non-small cell lung cancer: A nonrandomized controlled trial. JAMA Oncol. (2024) 10:932–40. doi: 10.1001/jamaoncol.2024.1779 PMC12312504 38869865

[33] Zhang L Zlotoff DA Awadalla M Mahmood SS Nohria A Hassan MZO . Major adverse cardiovascular events and the timing and dose of corticosteroids in immune checkpoint inhibitor–associated myocarditis. Circulation. (2020) 141:2031–4. doi: 10.1161/CIRCULATIONAHA.119.044703 PMC7301778 32539614

[34] Verheijden RJ van Eijs MJM May AM van Wijk F Suijkerbuijk KPM . Immunosuppression for immune-related adverse events during checkpoint inhibition: an intricate balance. NPJ Precis Oncol. (2023) 7:41. doi: 10.1038/s41698-023-00380-1 37173424 PMC10182067

[35] Liu Z Fan Y Guo J Bian N Chen D . Fulminant myocarditis caused by immune checkpoint inhibitor: a case report and possible treatment inspiration. ESC Heart Failure. (2022) 9:2020–6. doi: 10.1002/ehf2.13912 PMC9065848 35322589

[36] Eftekhar SP Yazdanpanah N Rezaei N . Immune checkpoint inhibitors and cardiotoxicity: possible mechanisms, manifestations, diagnosis and management. Expert Rev Anticancer Ther. (2021) 21:1211–28. doi: 10.1080/14737140.2021.1979396 34511008

[37] Verheijden RJ Burgers FH Janssen JC Putker AE Veenstra S Hospers GAP . Corticosteroids and other immunosuppressants for immune-related adverse events and checkpoint inhibitor effectiveness in melanoma. Eur J Cancer. (2024) 207:114172. doi: 10.1016/j.ejca.2024.114172 38905818

[38] Eggermont AMM Kicinski M Blank CU Mandala M Long GV Atkinson V . Association between immune-related adverse events and recurrence-free survival among patients with stage III melanoma randomized to receive pembrolizumab or placebo: A secondary analysis of a randomized clinical trial. JAMA Oncol. (2020) 6:519–27. doi: 10.1001/jamaoncol.2019.5570 PMC6990933 31895407

[39] Faje AT Lawrence D Flaherty K Freedman C Fadden R Rubin K . High-dose glucocorticoids for the treatment of ipilimumab-induced hypophysitis is associated with reduced survival in patients with melanoma. Cancer. (2018) 124:3706–14. doi: 10.1002/cncr.31629 29975414

[40] Bai X Hu J Betof Warner A Quach HT Cann CG Zhang MZ . Early use of high-dose glucocorticoid for the management of irAE is associated with poorer survival in patients with advanced melanoma treated with anti-PD-1 monotherapy. Clin Cancer Res. (2021) 27:5993–6000. doi: 10.1158/1078-0432.CCR-21-1283 34376536 PMC9401488

[41] Draghi A Borch TH Radic HD Chamberlain CA Gokuldass A Svane IM . Differential effects of corticosteroids and anti-TNF on tumor-specific immune responses: implications for the management of irAEs. Int J Cancer. (2019) 145:1408–13. doi: 10.1002/ijc.v145.5 30575963

[42] Waliany S Lee D Witteles RM Neal JW Nguyen P Davis MM . Immune checkpoint inhibitor cardiotoxicity: understanding basic mechanisms and clinical characteristics and finding a cure. Annu Rev Pharmacol Toxicol. (2021) 61:113–34. doi: 10.1146/annurev-pharmtox-010919-023451 32776859

[43] Vicino A Hottinger AF Latifyan S Boughdad S Becce F Prior JO . Immune checkpoint inhibitor-related myositis and myocarditis: diagnostic pitfalls and imaging contribution in a real-world, institutional case series. J Neurol. (2023) 271:1947–58. doi: 10.1007/s00415-023-12134-x 38141128 PMC10973051

[44] Hamada N Maeda A Takase-Minegishi K Kirino Y Sugiyama Y Namkoong H . Incidence and distinct features of immune checkpoint inhibitor-related myositis from idiopathic inflammatory myositis: A single-center experience with systematic literature review and meta-analysis. Front Immunol. (2021) 12:803410. doi: 10.3389/fimmu.2021.803410 34938300 PMC8686164

[45] Nguyen LS Bretagne M Arrondeau J Zahr N Ederhy S Abbar B . Reversal of immune-checkpoint inhibitor fulminant myocarditis using personalized-dose-adjusted abatacept and ruxolitinib: proof of concept. J Immunother Cancer. (2022) 10:e004699. doi: 10.1136/jitc-2022-004699 35383117 PMC8984056

[46] Tajmir-Riahi A Bergmann T Schmid M Agaimy A Schuler G Heinzerling L . Life-threatening autoimmune cardiomyopathy reproducibly induced in a patient by checkpoint inhibitor therapy. J Immunother. (2018) 41:35–8. doi: 10.1097/CJI.0000000000000190 29077601

