# Supplementary material for: Efficacy of high-dose steroids versus low-dose steroids in the treatment of immune checkpoint inhibitor-associated myocarditis: a case series and systematic review
Source: Front Immunol. 2025 Feb 12;16:1455347. doi: 10.3389/fimmu.2025.1455347 (PMC11860070; doi:10.3389/fimmu.2025.1455347)

Supplementary figure 1: Treatment Course and Changes in cTnI and CK Levels in Case 1

Supplementary figure 2: Treatment Course and Changes in cTnI and CK Levels in Case 2

Supplementary figure 3: Treatment Course and Changes in cTnI and CK Levels in Case 3

Supplementary figure 4: Treatment Course and Changes in cTnI and CK Levels in Case 4

Supplementary figure 5: Treatment Course and Changes in cTnI Levels in Case 5

Supplementary figure 6: Selection Process of Research Reports Using Preferred Reporting Items for Systematic Reviews and Meta-Analyses (PRISMA) for Systematic Review and Meta-Analysis

Supplementary figure 1: Treatment Course and Changes in cTnI and CK Levels in Case 1


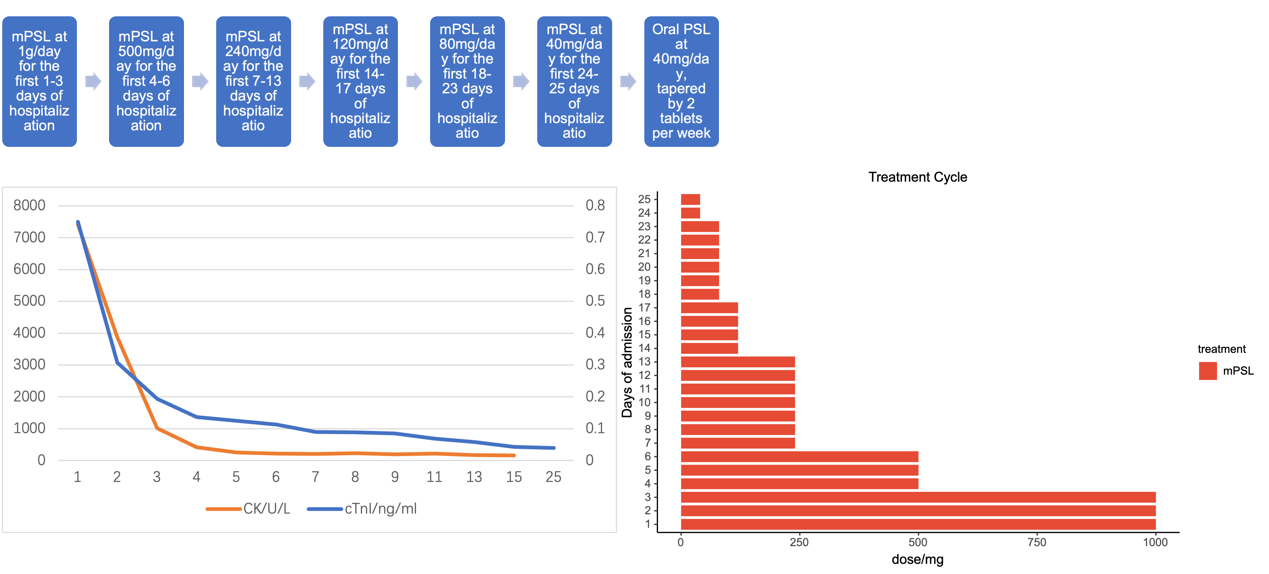


Supplementary figure 2: Treatment Course and Changes in cTnI and CK Levels in Case 2


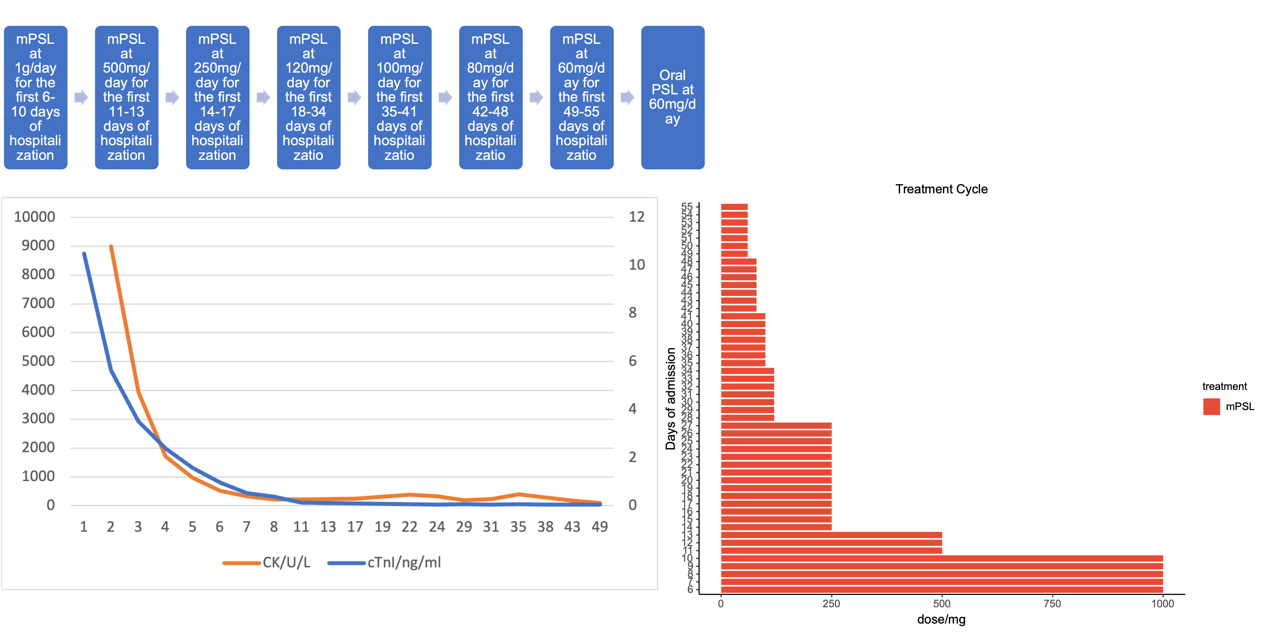


Supplementary figure 3: Treatment Course and Changes in cTnI and CK Levels in Case 3


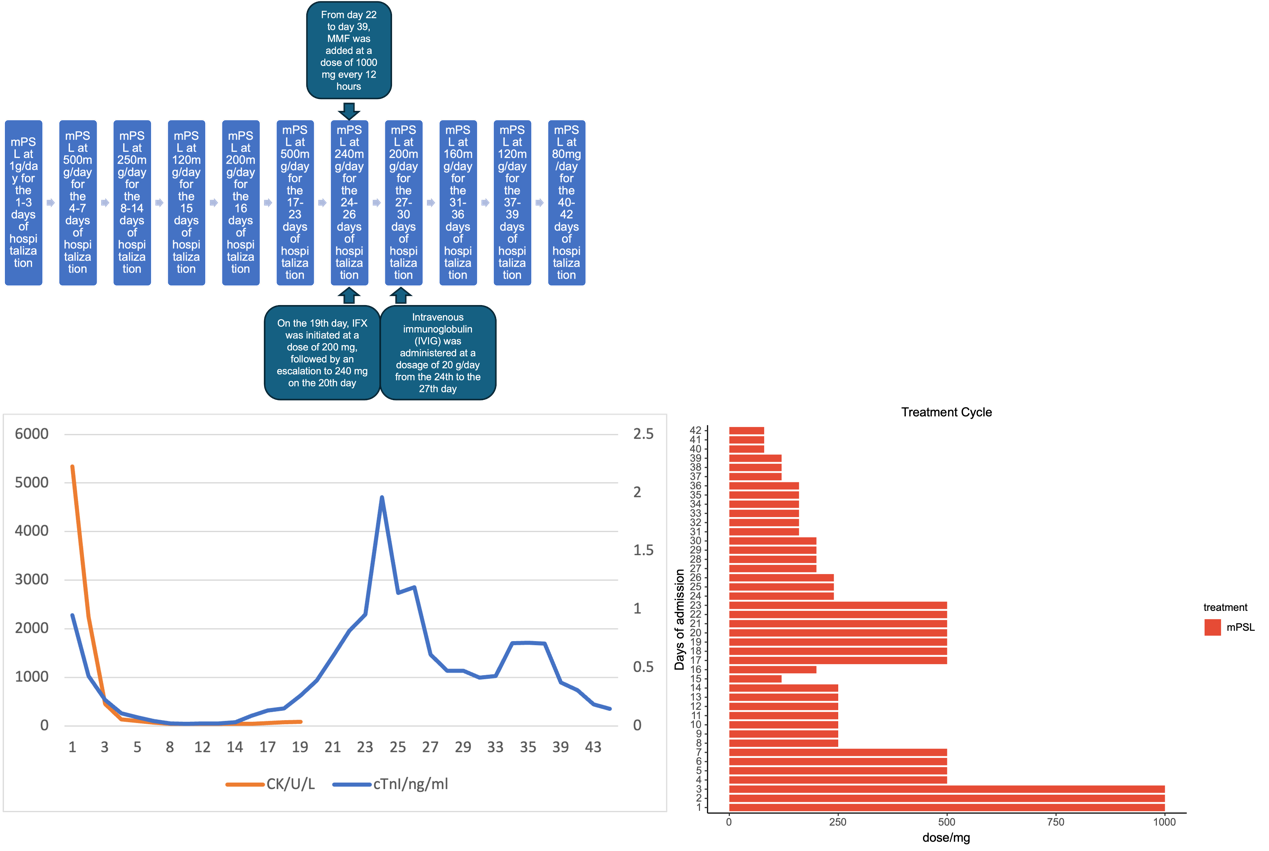


Supplementary figure 4: Treatment Course and Changes in cTnI and CK Levels in Case 4


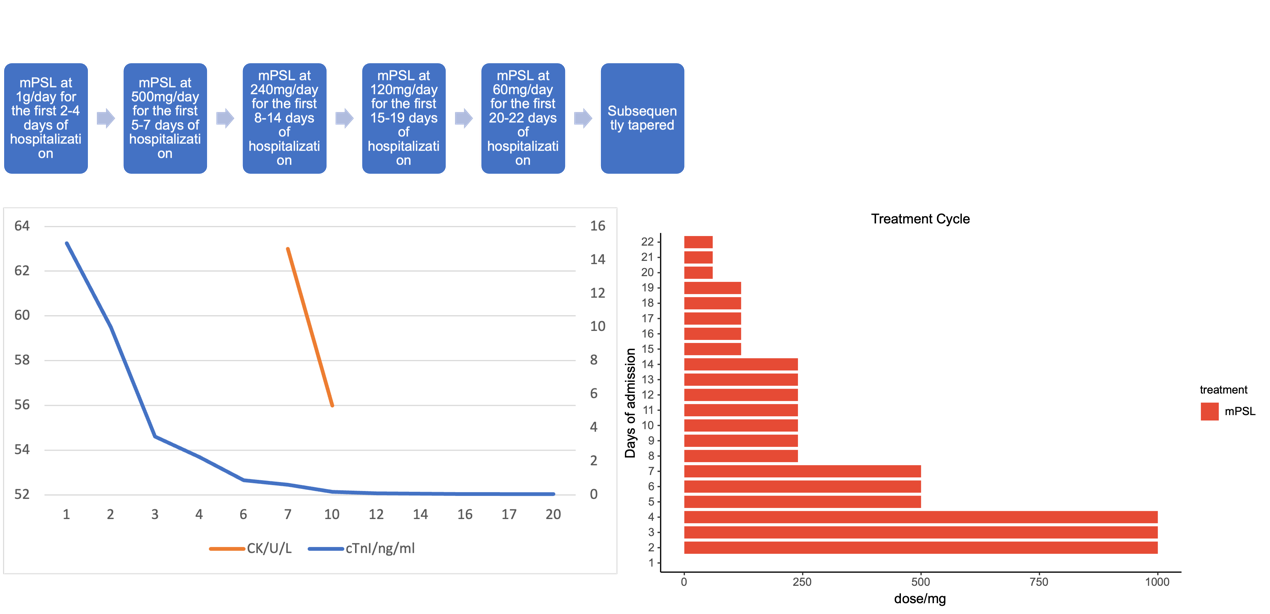


Supplementary figure 5: Treatment Course and Changes in cTnI Levels in Case 5


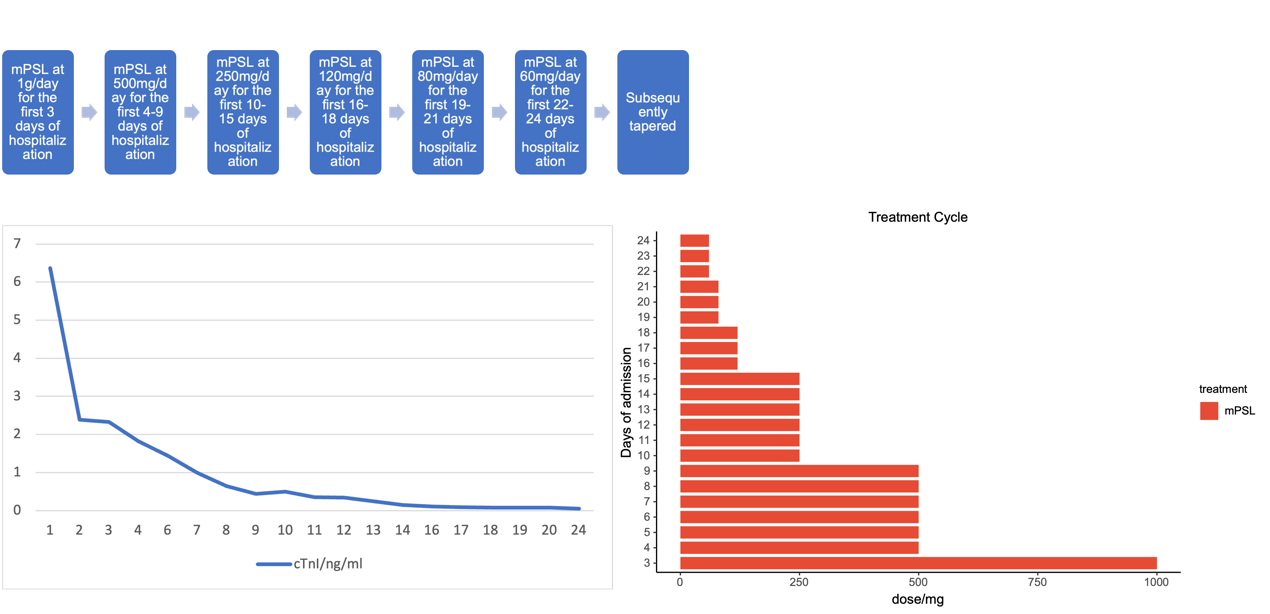


Supplementary figure 6: Ethical Committee Review Opinion


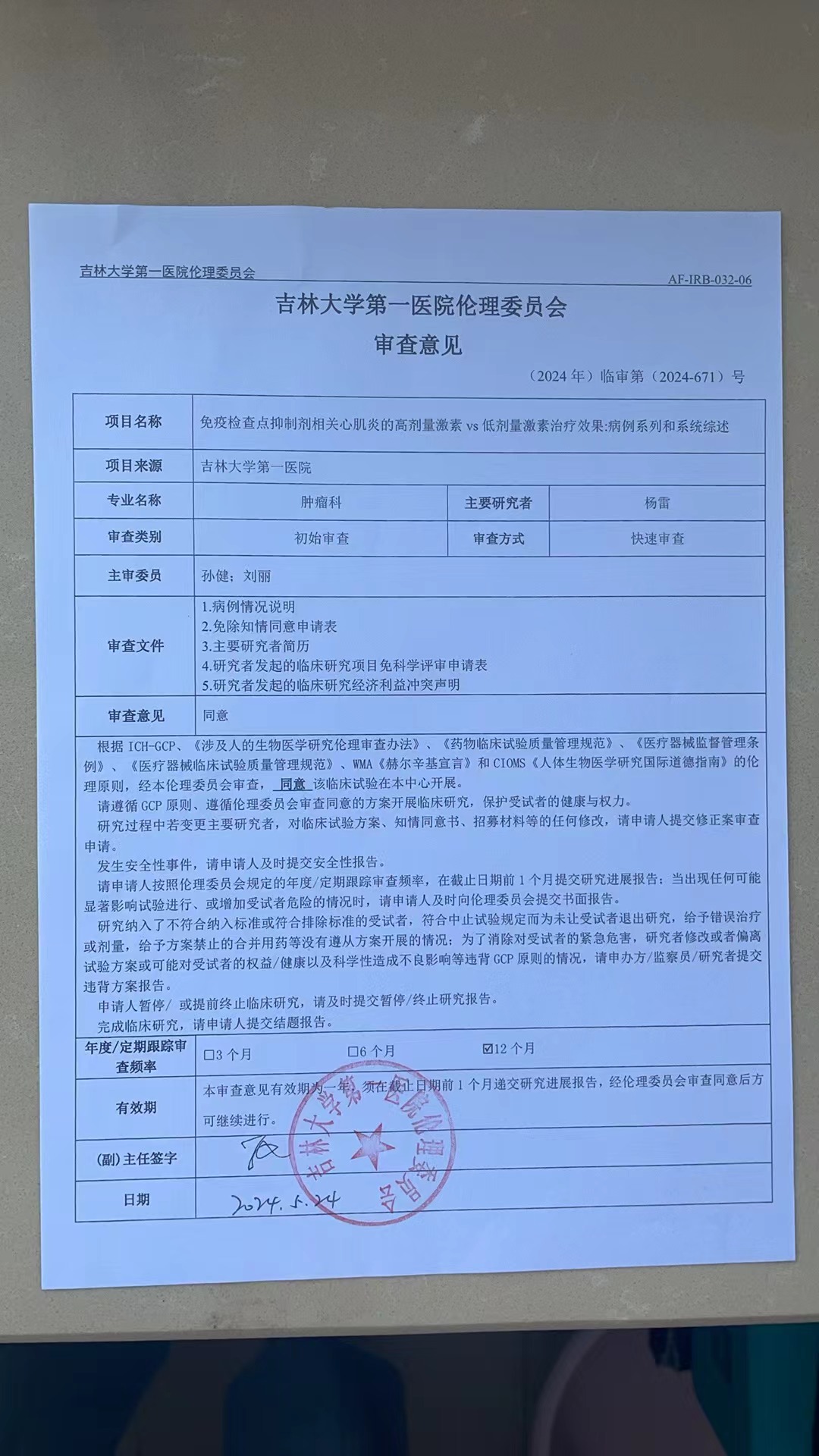

Supplement: Supplementary file 1 [file DataSheet1.docx]
