# Supplementary material for: Efficacy of high-dose steroids versus low-dose steroids in the treatment of immune checkpoint inhibitor-associated myocarditis: a case series and systematic review
Source: Front Immunol. 2025 Feb 12;16:1455347. doi: 10.3389/fimmu.2025.1455347 (PMC11860070; doi:10.3389/fimmu.2025.1455347)
Supplement: Supplementary file 2 [file DataSheet2.docx]

**Supplementary table 1: PubMed Embase and Cochrane library session results**

**Supplementary table 2: Statistical Information of High-Dose Patients Identified through Systematic Search**

**Supplementary table 3: Statistical Information of Low-Dose Patients Identified through Systematic Search**

**Supplementary table 4: General Evolution of cTnI/T, CK/NT-proBNP in Cases of High-Dose Group**

**Supplementary table 5: General Evolution of cTnI/T, CK/NT-proBNP in Cases of Low-Dose Group**

**Supplementary table 1: PubMed Embase and Cochrane library session results**

**PubMed session results**

| **Search number** | **Query** | **Sort By** | **Filters** | **Search Details** | **Results** |
| --- | --- | --- | --- | --- | --- |
| 7 | (("Immune Checkpoint Inhibitors"[Mesh]) OR ("Checkpoint Inhibitors, Immune" OR "Immune Checkpoint Inhibitor" OR "Checkpoint Inhibitor, Immune" OR "Immune Checkpoint Blockers" OR "Checkpoint Blockers, Immune" OR "Immune Checkpoint Blockade" OR "Checkpoint Blockade, Immune" OR "Immune Checkpoint Inhibition" OR "Checkpoint Inhibition, Immune" OR "PD-L1 Inhibitors" OR "PD L1 Inhibitors" OR "PD-L1 Inhibitor" OR "PD L1 Inhibitor" OR "Programmed Death-Ligand 1 Inhibitors" OR "Programmed Death Ligand 1 Inhibitors" OR "PD-1-PD-L1 Blockade" OR "Blockade, PD-1-PD-L1" OR "PD 1 PD L1 Blockade" OR "CTLA-4 Inhibitors" OR "CTLA 4 Inhibitors" OR "CTLA-4 Inhibitor" OR "CTLA 4 Inhibitor" OR "Cytotoxic T-Lymphocyte-Associated Protein 4 Inhibitors" OR "Cytotoxic T Lymphocyte Associated Protein 4 Inhibitors" OR "Cytotoxic T-Lymphocyte-Associated Protein 4 Inhibitor" OR "Cytotoxic T Lymphocyte Associated Protein 4 Inhibitor" OR "PD-1 Inhibitors" OR "PD 1 Inhibitors" OR "PD-1 Inhibitor" OR "Inhibitor, PD-1" OR "PD 1 Inhibitor" OR "Programmed Cell Death Protein 1 Inhibitor" OR "Programmed Cell Death Protein 1 Inhibitors")) AND (("Myocarditis"[Mesh]) OR (Carditis)) | | | ("Immune Checkpoint Inhibitors"[MeSH Terms] OR ("checkpoint inhibitors immune"[All Fields] OR "Immune Checkpoint Inhibitor"[All Fields] OR "checkpoint inhibitor immune"[All Fields] OR "Immune Checkpoint Blockers"[All Fields] OR "checkpoint blockers immune"[All Fields] OR "Immune Checkpoint Blockade"[All Fields] OR "checkpoint blockade immune"[All Fields] OR "Immune Checkpoint Inhibition"[All Fields] OR "checkpoint inhibition immune"[All Fields] OR "pd l1 inhibitors"[All Fields] OR "pd l1 inhibitors"[All Fields] OR "pd l1 inhibitor"[All Fields] OR "pd l1 inhibitor"[All Fields] OR "programmed death ligand 1 inhibitors"[All Fields] OR "programmed death ligand 1 inhibitors"[All Fields] OR "pd 1 pd l1 blockade"[All Fields] OR "blockade pd 1 pd l1"[All Fields] OR "pd 1 pd l1 blockade"[All Fields] OR "ctla 4 inhibitors"[All Fields] OR "ctla 4 inhibitors"[All Fields] OR "ctla 4 inhibitor"[All Fields] OR "ctla 4 inhibitor"[All Fields] OR "cytotoxic t lymphocyte associated protein 4 inhibitors"[All Fields] OR "cytotoxic t lymphocyte associated protein 4 inhibitors"[All Fields] OR "cytotoxic t lymphocyte associated protein 4 inhibitor"[All Fields] OR "cytotoxic t lymphocyte associated protein 4 inhibitor"[All Fields] OR "pd 1 inhibitors"[All Fields] OR "pd 1 inhibitors"[All Fields] OR "pd 1 inhibitor"[All Fields] OR "inhibitor pd 1"[All Fields] OR "pd 1 inhibitor"[All Fields] OR "Programmed Cell Death Protein 1 Inhibitor"[All Fields] OR "Programmed Cell Death Protein 1 Inhibitors"[All Fields])) AND ("Myocarditis"[MeSH Terms] OR ("Myocarditis"[MeSH Terms] OR "Myocarditis"[All Fields] OR "carditis"[All Fields])) | 592 |
| 6 | ("Myocarditis"[Mesh]) OR (Carditis) | | | "Myocarditis"[MeSH Terms] OR "Myocarditis"[MeSH Terms] OR "Myocarditis"[All Fields] OR "carditis"[All Fields] | 28,412 |
| 5 | Carditis |  |  | "myocarditis"[MeSH Terms] OR "myocarditis"[All Fields] OR "carditis"[All Fields] | 28,412 |
| 4 | "Myocarditis"[Mesh] | Most Recent |  | "Myocarditis"[MeSH Terms] | 17,567 |
| 3 | ("Immune Checkpoint Inhibitors"[Mesh]) OR ("Checkpoint Inhibitors, Immune" OR "Immune Checkpoint Inhibitor" OR "Checkpoint Inhibitor, Immune" OR "Immune Checkpoint Blockers" OR "Checkpoint Blockers, Immune" OR "Immune Checkpoint Blockade" OR "Checkpoint Blockade, Immune" OR "Immune Checkpoint Inhibition" OR "Checkpoint Inhibition, Immune" OR "PD-L1 Inhibitors" OR "PD L1 Inhibitors" OR "PD-L1 Inhibitor" OR "PD L1 Inhibitor" OR "Programmed Death-Ligand 1 Inhibitors" OR "Programmed Death Ligand 1 Inhibitors" OR "PD-1-PD-L1 Blockade" OR "Blockade, PD-1-PD-L1" OR "PD 1 PD L1 Blockade" OR "CTLA-4 Inhibitors" OR "CTLA 4 Inhibitors" OR "CTLA-4 Inhibitor" OR "CTLA 4 Inhibitor" OR "Cytotoxic T-Lymphocyte-Associated Protein 4 Inhibitors" OR "Cytotoxic T Lymphocyte Associated Protein 4 Inhibitors" OR "Cytotoxic T-Lymphocyte-Associated Protein 4 Inhibitor" OR "Cytotoxic T Lymphocyte Associated Protein 4 Inhibitor" OR "PD-1 Inhibitors" OR "PD 1 Inhibitors" OR "PD-1 Inhibitor" OR "Inhibitor, PD-1" OR "PD 1 Inhibitor" OR "Programmed Cell Death Protein 1 Inhibitor" OR "Programmed Cell Death Protein 1 Inhibitors") | | | "Immune Checkpoint Inhibitors"[MeSH Terms] OR "checkpoint inhibitors immune"[All Fields] OR "Immune Checkpoint Inhibitor"[All Fields] OR "checkpoint inhibitor immune"[All Fields] OR "Immune Checkpoint Blockers"[All Fields] OR "checkpoint blockers immune"[All Fields] OR "Immune Checkpoint Blockade"[All Fields] OR "checkpoint blockade immune"[All Fields] OR "Immune Checkpoint Inhibition"[All Fields] OR "checkpoint inhibition immune"[All Fields] OR "pd l1 inhibitors"[All Fields] OR "pd l1 inhibitors"[All Fields] OR "pd l1 inhibitor"[All Fields] OR "pd l1 inhibitor"[All Fields] OR "programmed death ligand 1 inhibitors"[All Fields] OR "programmed death ligand 1 inhibitors"[All Fields] OR "pd 1 pd l1 blockade"[All Fields] OR "blockade pd 1 pd l1"[All Fields] OR "pd 1 pd l1 blockade"[All Fields] OR "ctla 4 inhibitors"[All Fields] OR "ctla 4 inhibitors"[All Fields] OR "ctla 4 inhibitor"[All Fields] OR "ctla 4 inhibitor"[All Fields] OR "cytotoxic t lymphocyte associated protein 4 inhibitors"[All Fields] OR "cytotoxic t lymphocyte associated protein 4 inhibitors"[All Fields] OR "cytotoxic t lymphocyte associated protein 4 inhibitor"[All Fields] OR "cytotoxic t lymphocyte associated protein 4 inhibitor"[All Fields] OR "pd 1 inhibitors"[All Fields] OR "pd 1 inhibitors"[All Fields] OR "pd 1 inhibitor"[All Fields] OR "inhibitor pd 1"[All Fields] OR "pd 1 inhibitor"[All Fields] OR "Programmed Cell Death Protein 1 Inhibitor"[All Fields] OR "Programmed Cell Death Protein 1 Inhibitors"[All Fields] | 26,877 |
| 2 | "Checkpoint Inhibitors, Immune" OR "Immune Checkpoint Inhibitor" OR "Checkpoint Inhibitor, Immune" OR "Immune Checkpoint Blockers" OR "Checkpoint Blockers, Immune" OR "Immune Checkpoint Blockade" OR "Checkpoint Blockade, Immune" OR "Immune Checkpoint Inhibition" OR "Checkpoint Inhibition, Immune" OR "PD-L1 Inhibitors" OR "PD L1 Inhibitors" OR "PD-L1 Inhibitor" OR "PD L1 Inhibitor" OR "Programmed Death-Ligand 1 Inhibitors" OR "Programmed Death Ligand 1 Inhibitors" OR "PD-1-PD-L1 Blockade" OR "Blockade, PD-1-PD-L1" OR "PD 1 PD L1 Blockade" OR "CTLA-4 Inhibitors" OR "CTLA 4 Inhibitors" OR "CTLA-4 Inhibitor" OR "CTLA 4 Inhibitor" OR "Cytotoxic T-Lymphocyte-Associated Protein 4 Inhibitors" OR "Cytotoxic T Lymphocyte Associated Protein 4 Inhibitors" OR "Cytotoxic T-Lymphocyte-Associated Protein 4 Inhibitor" OR "Cytotoxic T Lymphocyte Associated Protein 4 Inhibitor" OR "PD-1 Inhibitors" OR "PD 1 Inhibitors" OR "PD-1 Inhibitor" OR "Inhibitor, PD-1" OR "PD 1 Inhibitor" OR "Programmed Cell Death Protein 1 Inhibitor" OR "Programmed Cell Death Protein 1 Inhibitors" | | | "checkpoint inhibitors immune"[All Fields] OR "Immune Checkpoint Inhibitor"[All Fields] OR "checkpoint inhibitor immune"[All Fields] OR "Immune Checkpoint Blockers"[All Fields] OR "checkpoint blockers immune"[All Fields] OR "Immune Checkpoint Blockade"[All Fields] OR "checkpoint blockade immune"[All Fields] OR "Immune Checkpoint Inhibition"[All Fields] OR "checkpoint inhibition immune"[All Fields] OR "pd l1 inhibitors"[All Fields] OR "pd l1 inhibitors"[All Fields] OR "pd l1 inhibitor"[All Fields] OR "pd l1 inhibitor"[All Fields] OR "programmed death ligand 1 inhibitors"[All Fields] OR "programmed death ligand 1 inhibitors"[All Fields] OR "pd 1 pd l1 blockade"[All Fields] OR "blockade pd 1 pd l1"[All Fields] OR "pd 1 pd l1 blockade"[All Fields] OR "ctla 4 inhibitors"[All Fields] OR "ctla 4 inhibitors"[All Fields] OR "ctla 4 inhibitor"[All Fields] OR "ctla 4 inhibitor"[All Fields] OR "cytotoxic t lymphocyte associated protein 4 inhibitors"[All Fields] OR "cytotoxic t lymphocyte associated protein 4 inhibitors"[All Fields] OR "cytotoxic t lymphocyte associated protein 4 inhibitor"[All Fields] OR "cytotoxic t lymphocyte associated protein 4 inhibitor"[All Fields] OR "pd 1 inhibitors"[All Fields] OR "pd 1 inhibitors"[All Fields] OR "pd 1 inhibitor"[All Fields] OR "inhibitor pd 1"[All Fields] OR "pd 1 inhibitor"[All Fields] OR "Programmed Cell Death Protein 1 Inhibitor"[All Fields] OR "Programmed Cell Death Protein 1 Inhibitors"[All Fields] | 22,333 |
| 1 | "Immune Checkpoint Inhibitors"[Mesh] | Most Recent |  | "Immune Checkpoint Inhibitors"[MeSH Terms] | 10,030 |

# Embase session results

| **No.** | **Query** | **Results** |
| --- | --- | --- |
| #7 | #3 AND #6 | 1776 |
| #6 | #4 OR #5 | 51665 |
| #5 | 'myocarditis':ab,ti OR 'myocardial inflammation':ab,ti OR 'myocard inflammation':ab,ti OR 'inflammatory myocardiopathy':ab,ti OR 'inflammatory cardiomyopathy':ab,ti OR 'inflammation, myocardial':ab,ti OR 'cardiomyopathic inflammatory process':ab,ti OR 'cardiomyocyte inflammation':ab,ti | 35238 |
| #4 | 'myocarditis'/exp | 46216 |
| #3 | #1 OR #2 | 50499 |
| #2 | 'immune checkpoint blocker':ab,ti OR 'immune checkpoint inhibitors':ab,ti OR 'immune checkpoint inhibitor':ab,ti | 39043 |
| #1 | 'immune checkpoint inhibitor'/exp | 29988 |

**Cochrane library session results**

| **Search Name:** | |  |
| --- | --- | --- |
| **Date Run:** | **11/03/2024 10:37:38** | |
| **Comment:** |  |  |
|  |  |  |
| **ID** | **Search** | **Hits** |
| #1 | MeSH descriptor: [Immune Checkpoint Inhibitors] explode all trees | 256 |
| #2 | (“Checkpoint Inhibitors, Immune” or “Immune Checkpoint Inhibitor” or “Checkpoint Inhibitor, Immune” or “Immune Checkpoint Blockers” or “Checkpoint Blockers, Immune” or “Immune Checkpoint Blockade” or “Checkpoint Blockade, Immune” or “Immune Checkpoint Inhibition” or “Checkpoint Inhibition, Immune” or “PD L1 Inhibitors” or “PD L1 Inhibitor” or “Programmed Death Ligand 1 Inhibitors” or “PD 1 PD L1 Blockade” or “CTLA 4 Inhibitors” or “CTLA 4 Inhibitor” or “Cytotoxic T Lymphocyte Associated Protein 4 Inhibitors” or “Cytotoxic T Lymphocyte Associated Protein 4 Inhibitor” or “PD 1 Inhibitors” or “PD 1 Inhibitor” or “Programmed Cell Death Protein 1 Inhibitor” or “Programmed Cell Death Protein 1 Inhibitors”):ti,ab,kw | 2688 |
| #3 | #1 or #2 | 2688 |
| #4 | MeSH descriptor: [Myocarditis] explode all trees | 141 |
| #5 | (Myocarditis or Carditis):ti,ab,kw (Word variations have been searched) | 1429 |
| #6 | #4 or #5 | 1429 |
| #7 | #3 and #6 | 16 |

**Supplementary table 2: Statistical Information of High-Dose Patients Identified through Systematic Search**

| **Case** | **Age** | **Gender** | **Country** | **Tumor Type** | **Medical History** | **ICI** | **Time from initiation of ICI treatment to diagnosis of ICI-M (days)** | **Concomitant with other immune-related adverse events (irAEs)** | **Immunosuppressive Therapy (IST)** | **Clinical Symptoms** | **Electrocardiogram (ECG)** | **Ejection Fraction (EF)** | **cTnI≥32ULN（ng/ml**  **）** | **cTnT≥32ULN（ng/ml**  **）** | **Elevation or sustained elevation of cTnI/T after mPSL treatment** | **Reinitiation of mPSL or escalation of mPSL dosage after mPSL treatment** | **MACE at 3 Months Post-Treatment** | **Outcome** |
| --- | --- | --- | --- | --- | --- | --- | --- | --- | --- | --- | --- | --- | --- | --- | --- | --- | --- | --- |
| 1 | 74^1^ | Male | Excluding China | Urothelial carcinoma | Absent | Atezolizumab | 56 days | Myositis, Respiratory failure | No | Dyspnea | Right bundle branch block | Normal | Absent | Yes | No | No | Cardiovascular death | Cardiovascular death |
| 2 | 70^2^ | Male | Excluding China | Renal cell carcinoma | Absent | Ipilimumab, Nivolumab | Unknown | MG, Encephalitis | IVIG, Cardiac pacemaker | Dizziness | Atrioventricular conduction block | Normal | Absent | Yes | No | No | No | Survival |
| 3 | 68^3^ | Male | Excluding China | Melanoma | Hypertension, Prostate cancer Prostate cancer | Ipilimumab, Nivolumab | 56 days | Pituitary inflammation, Neuritis, Hepatitis | No | Tachypnea, Dermatitis, Diplopia | Cardiac arrhythmia | 75% | No | Absent | No | No | Cardiovascular death | Cardiovascular death |
| 4 | 73^4^ | Male | Japan | Non-small cell lung cancer | Smoking | Pembrolizumab | 37 days | Hepatitis | Cardiac pacemaker | Syncope, Fatigue | Atrioventricular conduction block | 70% | Absent | Unknown | No | No | No | Survival |
| 5 | 69^5^ | Male | Excluding China | Non-small cell lung cancer | Urothelial carcinoma | Pembrolizumab | 46 days | Hepatitis | No | Tachypnea | Anterolateral ST segment elevation | Normal | Absent | Yes | No | No | Absent | Survival |
| 6 | 71^6^ | Male | Excluding China | Non-small cell lung cancer | Atrial fibrillation, Diabetes mellitus, Hypertension, Chronic obstructive pulmonary disease | Durvalumab | 84 days | Absent | No | Dyspnea, Chest pain, Dizziness | Atrioventricular conduction block | 66% | Absent | No | No | No | No | Survival |
| 7 | 70^7^ | Male | Japan | Squamous Cell Carcinoma | Hypertension | Pembrolizumab | 56 days | Myositis, Renal injury | IVIG | Syncope, Weakness | Unknown | Unknown | Absent | Yes | No | No | No | Disease progression death |
| 8 | 41^8^ | Female | Excluding China | Melanoma | Hypothyroidism | Nivolumab, Ipilimumab | 90 days | Absent | Absent | Dyspnea | Supraventricular tachycardia | 15% | No | Absent | No | No | No | Survival |
| 9 | 30^9^ | Female | Excluding China | Non-small cell lung cancer | Absent | Pembrolizumab | 126 days | Absent | MMF, Cardiac pacemaker | Dyspnea | Supraventricular tachycardia | 18% | Absent | No | No | No | No | Survival |
| 10 | 84^10^ | Male | Excluding China | Melanoma | Hypertension, Asthma | Ipilimumab,Nivolumab | 20 days | MG, Hepatitis | Absent | Dyspnea | Atrioventricular conduction block | Unknown | Absent | Yes | No | No | Cardiovascular death | Cardiovascular death |
| 11 | 70^10^ | Male | Excluding China | Melanoma | Hypertension, Diabetes mellitus, Dyslipidemia | Ipilimumab, Nivolumab, relatlimab | 44 days | MG | Evolocumab, Cardiac pacemaker, IVIG, Plasma separation,abatacept | Absent | Supraventricular tachycardia | Unknown | Absent | No | Yes | No | No | Disease progression death |
| 12 | 60^11^ | Male | Excluding China | Melanoma | Absent | Nivolumab | 365 days | Absent | IVIG | Fatigue, Pyrexia | ST segment elevation | 70% | Absent | Yes | No | No | No | Survival |
| 13 | 57^12^ | Male | Excluding China | Renal cell carcinoma | Absent | Nivolumab | Unknown | Absent | Absent | Dyspnea | Supraventricular tachycardia | 32% | Yes | Absent | Yes | No | No | Survival |
| 14 | 69^13^ | Male | China | Thymoma | Hypertension, Hyperlipidemia, Atrial fibrillation, Graves | Pembrolizumab | 44 days | MG | IVIG, MMF | Dyspnea | Unknown | 48% | Yes | Absent | No | No | No | Disease progression death |
| 15 | 59^14^ | Male | Excluding China | Renal cell carcinoma | Absent | Ipilimumab, Nivolumab | 14 days | MG | Absent | Ptosis | Atrioventricular conduction block | Unknown | Yes | Absent | No | No | No | Survival |
| 16 | 54^15^ | Male | Excluding China | Esophageal adenocarcinoma | Absent | Nivolumab | 42 days | Absent | Absent | Dyspnea | Supraventricular tachycardia | 35% | Unknown | Absent | Unknown | No | Heart failure | Treatment abandonment death |
| 17 | 96^16^ | Male | Excluding China | Melanoma | Absent | Pembrolizumab | 21 days | Myositis, Hepatitis | IVIG | Dyspnea | Unknown | Unknown | Yes | Absent | No | No | No | Treatment abandonment death |
| 18 | 73^17^ | Female | Excluding China | Non-small cell lung cancer | Absent | Pembrolizumab | 42 days | Absent | Absent | Palpitations | Supraventricular tachycardia | Unknown | Yes | Absent | No | No | No | Survival |
| 19 | 77^18^ | Male | Excluding China | Melanoma | Absent | Ipilimumab, Nivolumab | 44 days | Absent | Absent | Absent | Abnormal T-wave | 40% | Yes | Absent | Unknown | No | No | Unknown |
| 20 | 45^18^ | Male | Excluding China | Melanoma | Absent | Ipilimumab, Nivolumab | 19 days | Absent | Absent | Absent | Supraventricular tachycardia | 40% | Yes | Absent | Unknown | No | No | Unknown |
| 21 | 77^19^ | Male | Excluding China | Melanoma | Hypertension, Diabetes mellitus, Psoriasis | Pembrolizumab | 28 days | Myositis, MG | IVIG, Rituximab | Tachypnea | Cardiac arrhythmia | Normal | Unknown | Unknown | Unknown | No | No | Survival |
| 22 | 75^20^ | Female | Excluding China | Primary peritoneal carcinoma | Absent | Pembrolizumab | 65 days | Absent | Absent | Pyrexia | Normal | 40-45% | No | Absent | No | No | No | Survival |
| 23 | 81^20^ | Female | Excluding China | Serous ovarian cancer | Absent | Durvalumab | 27 days | Absent | Absent | Dyspnea | Normal | Unknown | Yes | Absent | Yes | No | No | Treatment abandonment death |
| 24 | 57^21^ | Male | Excluding China | Small cell lung cancer | Absent | Ipilimumab, Nivolumab | 42 days | Myositis | tocilizumab | Dyspnea | ST segment elevation | Unknown | No | No | Yes | No | No | Survival |
| 25 | 77^22^ | Female | Japan | Non-small cell lung cancer | Absent | Nivolumab | 15 days | Myositis, MG | IVIG | Myalgia, Ptosis | ST segment elevation | Unknown | Absent | Yes | Yes | Yes | No | Respiratory failure death |
| 26 | 71^23^ | Female | Japan | Squamous Cell Carcinoma | Absent | Pembrolizumab | 21 days | Absent | Cardiac pacemaker | Palpitations | Atrial fibrillation | 60-65% | No | Absent | No | No | No | Survival |
| 27 | 47^24^ | Male | Excluding China | Neuroendocrine carcinoma | Absent | Nivolumab | 58 days | Absent | IVIG | Absent | Normal | Normal | Absent | Yes | Yes | No | No | Disease progression death |
| 28 | 77^25^ | Female | Japan | Melanoma | Absent | Ipilimumab, Nivolumab | 21 days | Absent | IVIG, MMF | Myalgia | Atrial fibrillation | Normal | Absent | No | Yes | No | No | Survival |
| 29 | 62^26^ | Male | Excluding China | Renal cell carcinoma | Absent | Ipilimumab, Nivolumab | 42 days | Myositis, MG | Plasma exchange | Dyspnea | Supraventricular tachycardia | 55% | Absent | Yes | No | No | No | Survival |
| 30 | 76^26^ | Male | Excluding China | Prostate cancer Prostate cancer | Coronary artery disease | Ipilimumab, Nivolumab | Unknown | Myositis, MG | Plasma exchange, Evolocumab, Rituximab | Dyspnea | Unknown | 63% | Absent | Yes | Yes | No | No | Survival |
| 31 | 72^26^ | Male | Excluding China | Inguinal squamous cell carcinoma | Absent | Tremelimumab | 42 days | Myositis, MG | Plasma exchange, IVIG, Rituximab | Lower limb muscle weakness | Ventricular premature beats | 64% | Absent | Yes | Yes | No | No | Survival |
| 32 | 72^26^ | Male | Excluding China | Non-small cell lung cancer | COVID-19 | Ipilimumab, Nivolumab | Unknown | Myositis, MG | Plasma exchange | Respiratory failure | Unknown | 40% | Absent | Yes | Yes | No | No | Survival |
| 33 | 79^27^ | Male | Excluding China | Pleural mesothelioma | Asbestos exposure, Hypertension, Dyslipidemia, Stage 3 chronic kidney disease | Nivolumab | 14 days | Myositis, MG | MMF | Muscle weakness, Dyspnea | Unknown | Unknown | Absent | Yes | Yes | No | No | Survival |
| 34 | 59^28^ | Female | China | Esophageal cancer | Absent | Camrelizumab | 42 days | Absent | IVIG | Absent | ST segment elevation | 62% | Absent | Yes | Yes | No | No | Survival |
| 35 | 60^29^ | Female | China | Cholangiocarcinoma | Diabetes mellitus | Camrelizumab | 42 days | Myositis, Acute liver injury | IVIG, MMF | Tachypnea, Muscle weakness | Normal | Normal | Yes | Absent | No | No | No | Survival |
| 36 | 65^30^ | Male | China | Rectal adenocarcinoma | Absent | Tislelizumab | 20 days | Myositis, MG, Liver injury, Renal injury | IVIG | Muscle weakness | Unknown | Unknown | Yes | Absent | No | No | No | Survival |
| 37 | 42^31^ | Male | China | Thymoma | Absent | Sintilimab | 15 days | Liver injury | IVIG | Pyrexia | Unknown | Unknown | Yes | Absent | Yes | No | No | Treatment abandonment death |
| 38 | 52^31^ | Male | China | Thymoma | MG | Tislelizumab | 23 days | Liver injury | IVIG, tocilizumab | Chest tightness | ST segment elevation | Unknown | Yes | Absent | Yes | No | No | Survival |
| 39 | 60^32^ | Male | China | Non-small cell lung cancer | Absent | Pembrolizumab | 36 days | Myositis | Absent | Tachypnea | ST segment elevation | Normal | Yes | Absent | No | No | No | Survival |
| 40 | 54^33^ | Male | Excluding China | Melanoma | Absent | Pembrolizumab, Ipilimumab | 35 days | Absent | Absent | Dyspnea | Conduction block | 35% | No | Absent | No | No | No | Survival |
| 41 | 25^34^ | Male | Excluding China | Thymoma | Absent | pembrolizumab | 14 days | Absent | Abatacept, MMF, Ramipril | Myalgia | Cardiac arrhythmia | 60% | Absent | Yes | Yes | No | No | Survival |
| 42 | 65^35^ | Female | Excluding China | Esophageal cancer | Hypertension, Hypercholesterolemia, Diabetes mellitus, Smoking | Nivolumab | Unknown | Absent | rATG, MMF | Dyspnea, Dizziness | Left bundle branch block, Ventricular tachycardia, Atrial fibrillation | 35% | Yes | Absent | No | No | No | Disease progression death |
| 43 | 70^36^ | Female | Japan | HCC | Hepatitis C | Atezolizumab | 4 days | Absent | Absent | Tachypnea, Fatigue | ST abnormality | 40% | Absent | Yes | Yes | No | No | Survival |

"Absent" denotes the absence of specific information in the literature. "Unknown" indicates that the literature mentions the item but lacks explicit details.

**Supplementary table 3: Statistical Information of Low-Dose Patients Identified through Systematic Search**

| **Case** | **Age** | **Gender** | **Country** | **Tumor Type** | **Medical History** | **ICI** | **Time from initiation of ICI treatment to diagnosis of ICI-M (days)** | **Dosage (mg/kg/d or mg/d)** | **Concomitant with other immune-related adverse events (irAEs)** | **Immunosuppressive Therapy (IST)** | **Clinical Symptoms** | **Electrocardiogram (ECG)** | **Ejection Fraction (EF)** | **I≥32ULN（ng/ml**  **）** | **T≥32ULN（ng/ml**  **）** | **Elevation or sustained elevation of cTnI/T after mPSL treatment** | **Reinitiation of mPSL or escalation of mPSL dosage after mPSL treatment** | **MACE at 3 Months Post-Treatment** | **Outcome** |
| --- | --- | --- | --- | --- | --- | --- | --- | --- | --- | --- | --- | --- | --- | --- | --- | --- | --- | --- | --- |
| 1 | 65^10^ | Female | Excluding China | Melanoma | Hypertension, Diabetes mellitus, Pancreatitis | Nivolumab, relatlimab | 37 days | 1mg/kg/d | MG | Plasma separation, Abatacept | Dyspnea | Unknown | Unknown | Absent | Yes | Yes | Yes | No | Survival |
| 2 | 75^19^ | Male | Excluding China | Melanoma | Hypertension, Hyperlipidemia, Smoking | Pembrolizumab | 35 days | 500mg/d | Myositis, MG | IVIG, Rifaximab | Myalgia | Conduction block | Unknown | Absent | Unknown | Unknown | No | No | Survival |
| 3 | 60^20^ | Female | Excluding China | Endometrial cancer | Absent | Pembrolizumab | 21 days | 2mg/kg/d | Absent | Absent | Fatigue | Normal | 25% | Absent | Yes | Unknown | No | Cardiovascular death | Cardiovascular death |
| 4 | 60^37^ | Female | Excluding China | Melanoma | Absent | Nivolumab | 58 days | 2mg/kg/d | Absent | Absent | Palpitations | ST segment elevation | 30.2% | Absent | Unknown | Unknown | No | No | Survival |
| 5 | 78^38^ | Female | Excluding China | Melanoma | Hypertension, Hypothyroidism | Pembrolizumab | 51 days | 2mg/kg/d | Absent | Absent | Chest pain | Non-ST-segment elevation myocardial infarction | Unknown | Absent | Yes | Yes | No | No | Survival |
| 6 | 67^39^ | Male | Excluding China | Melanoma | Absent | Ipilimumab, Nivolumab | 6 days | 1mg/kg/d | Dermatitis, Renal injury | ATG | Dyspnea | Ventricular conduction block | 60% | Absent | Yes | Yes | Yes | Cardiovascular death | Cardiovascular death |
| 7 | 80^40^ | Male | Excluding China | Renal cell carcinoma | Hypertension | Nivolumab | 60 days | 2mg/kg/d | MG | Absent | Weakness, Pain | Atrial fibrillation, Left bundle branch block | Unknown | Yes | Absent | No | No | Cardiovascular death | Cardiovascular death |
| 8 | 52^41^ | Female | Excluding China | Non-small cell lung cancer | Smoking, Chronic obstructive pulmonary disease, Asthma | Ipilimumab, Nivolumab | 365 days | 125mg/d | Absent | Absent | Dyspnea, | Sinus tachycardia | 15-20% | Yes | Absent | No | No | No | Survival |
| 9 | 67^42^ | Female | Excluding China | Urothelial carcinoma | Hypertension | Pembrolizumab | 84 days | 120mg/d | Absent | Absent | Fatigue, Palpitations, Pain | Right bundle branch block | 30% | Yes | Absent | No | No | Cardiovascular death | Cardiovascular death |
| 10 | 53^43^ | Female | Excluding China | Ovarian cancer | Absent | Pembrolizumab | 4 days | 1mg/kg/d | Liver injury | Evolocumab | Absent | Normal | 50% | Absent | No | Yes | Yes | Heart failure | Survival |
| 11 | 62^43^ | Female | Excluding China | Renal cell carcinoma | Absent | Nivolumab | 45 days | 1mg/kg/d | Absent | Evolocumab | Dyspnea | ST segment elevation | 25% | Absent | Yes | Unknown | Yes | Heart failure | Unknown |
| 12 | 70^44^ | Female | Excluding China | Thymoma | Absent | Pembrolizumab | 16 days | 1mg/kg/d | MG | Plasma separation, Cardiac pacemaker | Dyspnea | ST segment elevation | 63% | Absent | No | Yes | Yes | No | Survival |
| 13 | 70^45^ | Male | Excluding China | Non-small cell lung cancer | Coronary artery disease | Durvalumab | 49 days | 2mg/kg/d | MG | Absent | Dyspnea | Atrial fibrillation, Right bundle branch block | Normal | Absent | Yes | No | No | No | Survival |
| 14 | 60^46^ | Male | Excluding China | Non-small cell lung cancer | Absent | PD-1 | 21 days | 80mg/d | Myositis, Hepatitis | Cardiac pacemaker, Evolocumab | Weakness, Fatigue, Chest pain | Atrioventricular block | 58% | Yes | Absent | Yes | No | No | Survival |
| 15 | 66^47^ | Female | Caucasus | Melanoma | Diabetes mellitus, Hypertension, Hyperlipidemia, Hypothyroidism | Ipilimumab | 15 days | 1mg/kg/d | MG | No | Dyspnea | Normal | 61% | Absent | Yes | No | No | No | Survival |
| 16 | 68^47^ | Male | Caucasus | Melanoma | Absent | Nivolumab | 14 days | 1mg/kg/d | MG | IVIG | Ptosis | Unknown | 58% | Absent | Yes | No | No | No | Survival |
| 17 | 62^48^ | Male | Excluding China | Hepatocellular carcinoma | Hepatitis | Nivolumab | 21 days | 2mg/kg/d | Absent | Absent | Chest pain | Sinus rhythm, Right bundle branch block,Left anterior fascicular block | 18% | Absent | Yes | Unknown | No | No | Disease progression death |
| 18 | 72^49^ | Male | Caucasus | Non-small cell lung cancer | Prostate cancer Prostate cancer, Central serous chorioretinopathy,  Obstructive sleep apnea, Diabetes mellitus, Hypertension, Chronic obstructive pulmonary disease | Durvalumab | 18 days | 1mg/kg/d | MG, Myositis, Hepatitis | Plasma exchange | Tachypnea, Weakness | ST segment elevation | Unknown | Absent | Yes | Unknown | No | No | Unknown |
| 19 | 55^50^ | Male | Excluding China | Melanoma | Absent | Ipilimumab, Nivolumab | 28 days | 0.2mg/kg/d | MG | IVIG, MMF | Weakness | Abnormal | Normal | No | Absent | Yes | Yes | Heart failure | Survival |
| 20 | 55^51^ | Female | Excluding China | Melanoma | Hypertension | Pembrolizumab | 28 days | 16mg/d | MG | Abatacept, IVIG, Cardiac pacemaker, Plasma exchange | Weakness | ST segment elevation | 55% | Absent | Yes | Unknown | Yes | Heart failure | Survival |
| 21 | 74^52^ | Male | Excluding China | Non-small cell lung cancer | Hypertension, Cerebrovascular accident | Pembrolizumab | 40 days | 1mg/kg/d | Renal injury, Hepatitis | Absent | Dyspnea | ST segment elevation | 30% | Absent | Yes | Unknown | No | No | Disease progression death |
| 22 | 66^53^ | Male | Excluding China | Non-small cell lung cancer | Absent | Ipilimumab, Nivolumab | 28 days | 2mg/kg/d | Myositis | Absent | Dyspnea, Fatigue, Pain | Atrial fibrillation | Abnormal | Absent | Yes | No | No | No | Disease progression death |
| 23 | 43^54^ | Male | Excluding China | Colon cancer | Diabetes mellitus | Pembrolizumab | 210 days | 125mg/d | Pancreatitis | Absent | Dyspnea | Left bundle branch block | 15-20% | Absent | No | Unknown | No | No | Survival |
| 24 | 82^55^ | Female | Caucasus | Urothelial carcinoma | Hypertension, Diabetes mellitus | Pembrolizumab | 25 days | 1mg/kg/d | Myositis | Cardiac pacemaker | Myalgia | Atrioventricular conduction block | 70% | Absent | Yes | No | No | No | Survival |
| 25 | 70^56^ | Male | Excluding China | Melanoma | Hypertension, Colon cancer | Ipilimumab, Nivolumab | 11 days | 1mg/kg/d | MG, Myositis, Hepatitis | MMF | Palpitations | Supraventricular tachycardia, Right bundle branch block | Normal | Absent | Yes | Yes | Yes | Cardiovascular death | Cardiovascular death |
| 26 | 79^56^ | Male | Excluding China | Melanoma | Chronic lymphocytic leukemia | Pembrolizumab | 26 days | 1mg/kg/d | MG, Myositis, Hepatitis, Gastritis | ATG, MMF, Cardiac pacemaker, IVIG | Fatigue, Weakness, Pain | Unknown | 60-65% | Absent | Yes | No | Yes | No | Disease progression death |
| 27 | 61^56^ | Female | Excluding China | Breast cancer | Absent | Durvalumab,Tremelimumab | 28 days | 2mg/kg/d | MG, Myositis, Hepatitis | MMF | Ptosis | Unknown | 60-65% | Absent | No | Yes | Yes | Cardiovascular death | Cardiovascular death |
| 28 | 69^56^ | Male | Excluding China | Urothelial carcinoma | CKD, Hyperlipidemia, Hypertension, Diabetes mellitus | Pembrolizumab | 132 days | 1mg/kg/d | Cerebrovascular accident | MMF | Pain, Weakness | Non-ST-segment elevation myocardial infarction | 40-45% | Absent | Yes | Yes | Yes | Cardiovascular death | Cardiovascular death |
| 29 | 67^56^ | Female | Excluding China | Melanoma | Absent | Ipilimumab, Nivolumab | 14 days | 2mg/kg/d | MG, Myositis, Hepatitis | ATG | Weakness, Dyspnea | Unknown | 60-65% | Absent | Yes | Yes | Yes | Cardiovascular death | Cardiovascular death |
| 30 | 83^56^ | Male | Excluding China | Melanoma | Hyperlipidemia,Hypertension, Atrial fibrillation | Nivolumab | 31 days | 1mg/kg/d | MG, Myositis, Hepatitis | Plasma exchange | Fatigue, Weakness, Chest pain, Apnea | Unknown | 55% | Absent | No | No | No | Cardiovascular death | Cardiovascular death |
| 31 | 70^56^ | Male | Excluding China | Renal cell carcinoma | Hypertension, Atrial fibrillation, CKD | Ipilimumab, Nivolumab | 21 days | 0.5mg/kg/d | MG, Myositis, Hepatitis, Gastritis | Evolocumab | Weakness, Fatigue | Cardiac arrhythmia | 60-65% | Absent | Yes | Unknown | Yes | No | Respiratory failure resulting in death |
| 32 | 89^56^ | Male | Excluding China | Non-small cell lung cancer | Hypertension, Hyperlipidemia, Coronary artery disease, CKD, Diabetes mellitus | Pembrolizumab | 32 days | 1mg/kg/d | MG, Myositis, Hepatitis | Absent | Dysphagia | Atrioventricular conduction block | 47% | Absent | Yes | Yes | No | No | Treatment abandonment death |
| 33 | 45^57^ | Female | China | Gastric carcinoma | Absent | Sintilimab | 21 days | 120mg/d | Absent | Absent | Chest pain, Palpitations | ST segment elevation | 68% | Yes | Absent | No | No | No | Survival |
| 34 | 64^58^ | Female | Excluding China | Glioblastoma multiforme | Absent | Nivolumab | 22 days | 500mg/d | Absent | Evolocumab, ATG, MMF | Weakness | Unknown | 37% | Yes | Absent | Unknown | No | No | Survival |
| 35 | 66^59^ | Female | Excluding China | Melanoma | Absent | Ipilimumab, Nivolumab | 112 days | 1mg/kg/d | Pneumonia | solumedrol | Dyspnea,Pain | Sinus tachycardia | Unknown | No | Absent | No | No | No | Survival |
| 36 | 73^60^ | Male | Excluding China | Hepatocellular carcinoma | Absent | Nivolumab | Unknown | 500mg/d | Absent | Absent | Absent | ST segment abnormality | 65% | No | Absent | Yes | No | No | Survival |
| 37 | 67^61^ | Male | Excluding China | Squamous Cell Carcinoma | Thymoma | Pembrolizumab | 14 days | 250mg/d | Myositis, MG | Plasma exchange | Dyspnea | Unknown | Unknown | Unknown | Absent | Yes | Yes | No | Respiratory failure resulting in death |
| 38 | 78^62^ | Female | Excluding China | Melanoma | Hypertension, Asthma, Pulmonary embolism, Major Depressive Disorder | Ipilimumab, Nivolumab | 5 days | 1mg/kg/d | Myositis, MG | Plasma exchange, IVIG | Myalgia, Weakness | Unknown | Unknown | Yes | Absent | No | Yes | No | Respiratory failure resulting in death |
| 39 | 88^63^ | Male | Excluding China | Melanoma | Absent | Nivolumab | 22 days | 40mg/d | Myositis | Cardiac pacemaker, Evolocumab | Absent | Normal | Normal | Absent | Yes | Yes | Yes | Cardiovascular death | Cardiovascular death |
| 40 | 45^64^ | Male | China | Nasopharyngeal carcinoma | Absent | Pembrolizumab | 4 days | 20mg/d | Pneumonia | Absent | Pyrexia, Myalgia, Dermatitis, Fatigue | ST segment elevation | Unknown | Absent | No | Yes | Yes | No | Survival |
| 41 | 57^65^ | Male | Excluding China | Renal cell carcinoma | Absent | Ipilimumab, Nivolumab | 12 days | 2mg/kg/d | Myositis, MG | MMF, Abatacept, Cardiac pacemaker, | Headache, Myalgia | Atrioventricular block, Right bundle branch block | 50% | No | Absent | Yes | Yes | Cardiac arrest | Disease progression death |
| 42 | 65^66^ | Female | Excluding China | Melanoma | Hypertension | Ipilimumab, Nivolumab | 12 days | 2mg/kg/d | Myositis, MG | Absent | Chest pain, Dyspnea, Fatigue | Atrioventricular block | 73% | Yes | Absent | Yes | No | Cardiovascular death | Cardiovascular death |
| 43 | 75^67^ | Female | Excluding China | Melanoma | Hypertension | Nivolumab | 175 days | 40mg/d | Absent | Absent | Fatigue, Edema, Pruritus | Unknown | Unknown | Absent | No | Yes | No | No | Survival |
| 44 | 74^68^ | Female | Excluding China | Non-small cell lung cancer | Absent | Ipilimumab, Nivolumab | 180 days | 2mg/kg/d | Absent | Absent | Fatigue, Palpitations | ST segment elevation | 50% | No | Absent | No | No | No | Disease progression death |
| 45 | 49^69^ | Female | Caucasus | Melanoma | Absent | Ipilimumab, Nivolumab | 20 days | 125mg/d | Absent | IVIG | Nausea | ST-segment changes | 55% | No | Absent | Yes | Yes | No | Survival |
| 46 | 75^70^ | Male | Excluding China | Renal cell carcinoma | Absent | Ipilimumab, Nivolumab | 53 days | 5mg/d | MG | Absent | Pain | Sinus arrhythmia | 59% | Yes | Absent | Yes | Yes | No | Unknown |
| 47 | 45^71^ | Female | China | Thymoma | Absent | Sintilimab | 16 days | 120mg/d | MG | IVIG, Plasma exchange | Abdominal pain, Chest tightness, Dizziness | Right bundle branch block | 71% | No | Absent | Yes | No | Cardiovascular death | Cardiovascular death |
| 48 | 71^72^ | Male | China | Hepatocellular carcinoma | Diabetes mellitus | Toripalimab | 27 days | 2mg/kg/d | Absent | Absent | Absent | Right bundle branch block, Left anterior fascicular block | 77% | No | Absent | Yes | No | No | Survival |
| 49 | 66^72^ | Male | China | Urothelial carcinoma | Absent | Tislelizumab | Unknown | 2mg/kg/d | Myositis, Hepatic injury, Renal injury | IVIG | Chest tightness | Right bundle branch block, Atrioventricular conduction block | Normal | Yes | Absent | Yes | Yes | No | Survival |
| 50 | 78^73^ | Male | China | Hepatocellular carcinoma | Hypertension | Sintilimab | 21 days | 2mg/kg/d | Absent | IVIG | Absent | Normal | Normal | No | Absent | Yes | Yes | No | Unknown |
| 51 | 67^74^ | Male | Excluding China | Melanoma | Hypertension, Hypercholesterolemia | Ipilimumab, Nivolumab | 16 days | 200mg/d | Myositis | Evolocumab | Fatigue, Weakness, Dyspnea | Atrioventricular conduction block | Normal | Unknown | Absent | Yes | Yes | Cardiovascular death | Cardiovascular death |
| 52 | 69^75^ | Male | Excluding China | Hepatocellular carcinoma | Absent | Pembrolizumab | 26 days | 1mg/kg/d | Myositis | Plasma exchange | Fatigue, Myalgia | Cardiac arrhythmia | 78% | Yes | Absent | Yes | Yes | Cardiovascular death | Cardiovascular death |
| 53 | 45^76^ | Female | Excluding China | Thymoma | Hepatitis | Pembrolizumab | 15 days | 2mg/kg/d | MG, Acute liver injury | Absent | Dyspnea, Weakness | Unknown | 56% | Yes | Absent | Yes | Yes | No | Treatment abandonment death |
| 54 | 71^77^ | Female | China | Extrahepatic cholangiocarcinoma | Biliary obstruction | Sintilimab | 30 days | 500mg/d | Absent | IVIG | Fatigue, Lumbar pain | Cardiac arrhythmia | 65% | Yes | Absent | No | No | No | Survival |
| 55 | 47^78^ | Female | China | Thymoma | Absent | Toripalimab | 30 days | 500mg/d | Myositis, MG | IVIG | Myalgia, Dyspnea, Weakness | Sinus tachycardia, Right bundle branch block | Unknown | Yes | Absent | Yes | No | No | Survival |
| 56 | 80^79^ | Female | China | Squamous cell carcinoma of the bone | Absent | Pembrolizumab | 21 days | 1mg/kg/d | Absent | Absent | Absent | Atrioventricular conduction block | Unknown | Yes | Absent | No | No | No | Survival |
| 57 | 67^80^ | Male | China | Large cell neuroendocrine carcinoma of the lung | Absent | Pembrolizumab | 14 days | 1mg/kg/d | MG, Liver injury | Absent | Dyspnea, Limb weakness | Left bundle branch block | 50% | Yes | Absent | No | No | No | Survival |
| 58 | 65^80^ | Male | Excluding China | Melanoma | Absent | Pembrolizumab | 12 days | 1mg/kg/d | MG, Myositis | Absent | Pyrexia, Myalgia | Atrial fibrillation | 60% | Yes | Absent | Unknown | No | No | Respiratory failure resulting in death |
| 59 | 67^81^ | Male | China | Non-small cell lung cancer | Absent | Sintilimab | Unknown | 240mg/d | Myositis | Absent | Dyspnea, Pyrexia, Myalgia | Supraventricular tachycardia | Unknown | No | Absent | Yes | No | No | Survival |
| 60 | 74^82^ | Female | Excluding China | Melanoma | Absent | PD-1 | Unknown | 2mg/kg/d | Myositis | MMF | Pain, Fatigue | Normal | Normal | No | Absent | No | No | No | Survival |
| 61 | 67^83^ | Male | China | Squamous Cell Carcinoma | Absent | Durvalumab | 70 days | 40mg/d | Absent | Absent | Pyrexia, Chest pain, Dyspnea | Sinus tachycardia | 41% | No | Absent | No | No | No | Survival |
| 62 | 68^84^ | Male | China | Non-small cell lung cancer | Absent | Sintilimab | 6 days | 80mg/d | Absent | Absent | Cough,Dysphagia | Myocardial infarction | Unknown | Yes | Absent | Yes | No | No | Survival |
| 63 | 69^85^ | Male | China | Esophageal cancer | Diabetes mellitus | Camrelizumab | 21 days | 120mg/d | MG, Liver injury, Pneumonia | IVIG | Lower limb weakness and Pain | Sinus tachycardia | 70% | No | Absent | Yes | Yes | No | Survival |
| 64 | 68^86^ | Female | China | Thymoma | Absent | Camrelizumab | 11 days | 80mg/d | Myositis, Hepatitis | IVIG, Cardiac pacemaker | Dyspnea, Fatigue, Muscle weakness, Palpitations | Atrioventricular conduction block | 65% | Yes | Absent | Yes | Yes | Cardiovascular death | Cardiovascular death |
| 65 | 48^87^ | Female | Excluding China | Thymoma | Absent | Pembrolizumab | 10 days | 2mg/kg/d | Absent | Evolocumab, Cardiac pacemaker | Dyspnea | Atrioventricular conduction block | 45-50% | Yes | Absent | Yes | Yes | Cardiovascular death | Cardiovascular death |
| 66 | 67^88^ | Male | Excluding China | Renal cell carcinoma | Absent | Ipilimumab, Nivolumab | 20 days | 1mg/kg/d | Absent | IVIG | Absent | Abnormal T-wave | Normal | Yes | Absent | No | Yes | Myocardial Infarction | Survival |
| 67 | 43^89^ | Female | China | Liposarcoma | Absent | Toripalimab | 42 days | 200mg/d | Myositis, Hashimoto’s thyroiditis, Rash | Absent | Chest pain, Palpitations, Dyspnea | Ventricular tachycardia | 55% | Yes | Yes | Yes | No | No | Survival |
| 68 | 64^89^ | Female | China | Pancreatic cancer | Hypertension | Toripalimab | 70 days | 200mg/d | Pneumonia | IVIG | Chest tightness | Right bundle branch block | 49% | Absent | Yes | Unknown | No | No | Survival |
| 69 | 67^90^ | Female | Excluding China | Urothelial carcinoma | Smoking | durvalumab | 336 days | 2mg/kg/d | Absent | MMF | Chest pain | ST segment elevation | 20-22% | Yes | Absent | Yes | Yes | Cardiovascular death | Cardiovascular death |
| 70 | 47^91^ | Female | Excluding China | Melanoma | Absent | Ipilimumab, Nivolumab | 7 days | 1mg/kg/d | Absent | IVIG | Absent | Normal | 55% | No | Absent | Yes | Yes | No | Survival |
| 71 | 81^92^ | Female | Excluding China | Melanoma | Hypertension, Dyslipidemia | Pembrolizumab, Ipilimumab, Nivolumab | 200 days | 1mg/kg/d | Absent | IVIG | Pyrexia, Dermatitis | Normal | Normal | No | Absent | Yes | Yes | No | Survival |
| 72 | 66^93^ | Female | Excluding China | Lung cancer | Absent | Nivolumab | 30 days | 500mg/d | Absent | Abatacept, Plasma exchange | Chest pain | Unknown | Unknown | Absent | Yes | Yes | No | No | Survival |
| 73 | 81^94^ | Male | Excluding China | Renal cell carcinoma | Absent | Ipilimumab, Nivolumab | 21 days | 500mg/d | Hepatitis | Plasma exchange | Fatigue | Atrioventricular conduction block | Unknown | Absent | Yes | Yes | Yes | Cardiovascular death | Cardiovascular death |
| 74 | 66^95^ | Female | China | Colorectal cancer | Hypertension, Diabetes mellitus | Sintilimab | 15 days | 500mg/d | Myositis, MG, Rash, Hepatitis | IVIG | Dermatitis, Fatigue, Muscle weakness, Dyspnea | Atrial Premature Contraction | 50% | Absent | No | Yes | No | No | Disease progression death |
| 75 | 69^96^ | Male | Excluding China | Unknown | Absent | Nivolumab | 11 days | 30mg/d | Myositis | Absent | Muscle weakness | Normal | Unknown | Absent | No | Yes | No | No | Disease progression death |
| 76 | 52^97^ | Male | China | Large Cell Neuroendocrine Carcinoma | Absent | Sintilimab | 120 days | 15mg/d | Absent | Absent | Tachypnea | Normal | Normal | Absent | Yes | Yes | Yes | No | Survival |
| 77 | 60^98^ | Male | China | Squamous Cell Carcinoma | Diabetes mellitus, Hypertension, Coronary artery disease | Sintilimab | 21 days | 1mg/kg/d | Absent | Absent | Absent | Normal | 67.6% | Absent | No | Yes | Unknown | No | Unknown |
| 78 | 72^99^ | Female | Excluding China | Melanoma | Basal cell carcinoma, Hypertension, Diabetes mellitus | Ipilimumab, Nivolumab | 35 days | 1.5mg/kg/d | Absent | Absent | Myalgia, Dyspnea | Normal | 63% | Absent | Yes | No | No | No | Disease progression death |
| 79 | 58^100^ | Female | Excluding China | Non-small cell lung cancer | Smoking | Pembrolizumab | 30 days | 1mg/kg/d | Absent | Absent | Dyspnea | Unknown | Unknown | Absent | Yes | Yes | No | Cardiovascular death | Cardiovascular death |
| 80 | 67^101^ | Male | China | Nasopharyngeal carcinoma | Absent | Toripalimab | 69 days | 4mg/kg/d | Absent | IVIG, Tofacitinib | Absent | Abnormal | 40% | Absent | No | Yes | Yes | No | Survival |
| 81 | 59^102^ | Female | Excluding China | Thymoma | Absent | Nivolumab | 14 days | 1mg/kg/d | Absent | Absent | Dyspnea | Ventricular tachycardia | 20-42% | Absent | Yes | Yes | Yes | Cardiovascular death | Cardiovascular death |
| 82 | 47^103^ | Female | Excluding China | Breast cancer | Thymoma | Toripalimab | 120 days | 2mg/kg/d | MG | Absent | Quadriplegia, Dysphagia, Pain | Unknown | Unknown | Absent | No | No | No | No | Survival |
| 83 | 67^104^ | Female | China | Colorectal cancer | Diabetes mellitus | Camrelizumab | 34 days | 360mg/d | MG | IVIG | Chest tightness, Tachypnea | Atrioventricular conduction block, Atrial premature complex,  Ventricular premature complex | Unknown | Absent | Yes | No | No | Cardiovascular death | Cardiovascular death |
| 84 | 77^105^ | Male | China | Spinal tumor | Absent | Sintilimab | 21 days | 480mg/d | MG | Absent | Chest tightness, Dyspnea | Abnormal | 61% | Absent | Unknown | Yes | No | No | Survival |
| 85 | 69^106^ | Male | China | Hepatocellular carcinoma | Absent | Camrelizumab | 25 days | 320mg/d | Absent | Absent | Tachypnea | ST segment depression | 61.2% | Absent | No | Yes | No | Cardiovascular death | Cardiovascular death |
| 86 | 75^106^ | Male | China | Squamous cell carcinoma | Smoking | Camrelizumab | 28 days | 320mg/d | Myositis | Absent | Tachypnea | ST segment depression | 63% | Absent | Yes | No | No | No | Survival |
| 87 | 69^107^ | Male | Excluding China | Cholangiocarcinoma | Absent | Ipilimumab,Nivolumab | 28 days | 0.5mg/kg/d | Absent | Absent | Absent | Normal | 57% | Absent | No | Yes | Yes | Stroke | Survival |
| 88 | 75^108^ | Male | Excluding China | Melanoma | Absent | Nivolumab | 48 days | 1mg/kg/d | Hepatocellular carcinoma | Absent | Absent | Unknown | Unknown | Absent | No | Yes | Yes | No | Disease progression death |
| 89 | 77^109^ | Male | China | Cholangiocarcinoma | Absent | Sintilimab | 21 days | 480mg/d | MG, Myositis, Hepatitis | Absent | Chest tightness, Tachypnea, Ptosis | ST segment depression | 61% | Absent | Yes | Unknown | No | No | Treatment abandonment death |
| 90 | 69^109^ | Female | China | Non-small cell lung cancer | Hypertension | Camrelizumab | 20 days | 240mg/d | MG, Hepatitis, Hypothyroidism | IVIG | Palpitations | Sinus tachycardia | 59% | Absent | Yes | No | Yes | No | Respiratory failure resulting in death |
| 91 | 69^110^ | Male | China | Gastric carcinoma | Absent | Pembrolizumab | 14 days | 500mg/d | Dermatitis, Myositis, Hepatitis | IVIG, Plasma exchange | Weakness, Tachypnea | ST segment elevation | 30% | Absent | Yes | Yes | No | No | Survival |
| 92 | 75^111^ | Male | Excluding China | Mesothelioma | Absent | Pembrolizumab | 21 days | 1.5mg/kg/d | MG, Hepatitis | Absent | Muscle weakness, Tachypnea, Ptosis | ST segment elevation | Unknown | Absent | Yes | Yes | No | No | Respiratory failure resulting in death |
| 93 | 51^112^ | Female | China | Breast cancer | Absent | PD-1 | 3 days | 80mg/d | Hepatitis | Absent | Pyrexia, Dyspnea, Dermatitis | Unknown | Unknown | Absent | No | Yes | No | No | Survival |
| 94 | 46^113^ | Female | China | Metastatic malignant neoplasm involving the supraclavicular lymph nodes | Absent | Pembrolizumab | 210 days | 1mg/kg/d | Absent | IVIG, Tofacitinib | Dyspnea, Palpitations | Normal | Normal | Absent | No | Yes | No | No | Survival |
| 95 | 60^114^ | Male | China | Soft tissue sarcoma | Absent | Camrelizumab | 56 days | 500mg/d | Hypothyroidism | Absent | Pyrexia, Fatigue, Tachypnea | Atrial fibrillation | Unknown | Absent | Unknown | Unknown | No | No | Survival |
| 96 | 83^115^ | Male | Caucasus | Non-small cell lung cancer | Chronic obstructive pulmonary disease, Diabetes mellitus, Hypertension, Hypothyroidism, Prostate cancer Prostate cancer,Colorectal cancer | Pembrolizumab | 47 days | 200mg/d | Pneumonia | Absent | Tachypnea | Unknown | Unknown | Absent | Yes | Yes | Unknown | No | Treatment abandonment death |
| 97 | 58^116^ | Male | Excluding China | Non-small cell lung cancer | Absent | Nivolumab | 540 days | 0.5mg/kg/d | Hepatitis | Evolocumab | Fatigue, Edema | Unknown | Normal | Absent | Unknown | Unknown | Yes | No | Survival |
| 98 | 80^117^ | Male | Caucasus | Squamous Cell Carcinoma | Diabetes mellitus, Hypertension, Smoking | Nivolumab | 42 days | 1mg/kg/d | Absent | Absent | Weakness | Normal | Unknown | Absent | Unknown | Unknown | No | Cardiovascular death | Cardiovascular death |

"Absent" denotes the absence of specific information in the literature. "Unknown" indicates that the literature mentions the item but lacks explicit details.

**Supplementary table 4: General Evolution of cTnI/T, CK/NT-proBNP in Cases of High-Dose Group**

| **Case** | **The maximum value of cTnI before mPSL treatment** | **The maximum value of cTnT before mPSL treatment** | **The maximum value of CK before mPSL treatment** | **The maximum value of NT-proBNP before mPSL treatment** | **The number of days after initial mPSL treatment when cTnI decreases by ≥90%** | **The number of days after initial mPSL treatment when cTnT decreases by ≥90%** | **The number of days after initial mPSL treatment when CK decreases by ≥90%** | **The number of days after initial mPSL treatment when NT-proBNP decreases by ≥90%** |
| --- | --- | --- | --- | --- | --- | --- | --- | --- |
| 1^29^ | 4.0 ng/mL | Absent | 24196 U/L | 3596 pg/ml | ＞8 days | Absent | ＞4 days | Unkonw |
| 2^30^ | 0.973ng/ml | Absent | 11920 U/L | Absent | ＞7 days | Absent | Unkonw | Absent |
| 3^31^ | 1.4237 ng/mL | Absent | 7603 U/L | Absent | Absent | Absent | Absent | Absent |
| 4^32^ | 39.778ng/ml | Absent | 18325 U/L | 2293pg/ml | ＜21 days | Absent | ＜21 days | ＜21 days |
| 5^33^ | 0.307 ng/ml | Absent | 636 U/L | Absent | ＞8 days | Absent | ＞8 days | Absent |
| 6^26^ | Absent | 0.620 ng/ml | 449 U/L | 52 pg/ml | Absent | ＞10 days | ＞2 days | Absent |
| 7^26^ | Absent | 1.383 ng/ml | 2206 U/L | 2446 pg/ml | Absent | ＞15 days | ＞5 days | Absent |
| 8^26^ | Absent | 2.262 ng/ml | 558 U/L | 827 pg/ml | Absent | ＞20 days | ＞5 days | Absent |
| 9^26^ | Absent | 0.565 ng/ml | 486 U/L | 27832 pg/ml | Absent | ＞15 days | ＞3 days | Absent |
| 10^27^ | Absent | 1.072 ng/ml ng/ml | 6602 U/L | Absent | Absent | ＞29 days | ＜7 days | Absent |
| 11^28^ | 0.2259 ng/ml | 0.6656 ng/ml | Absent | 1545 pg/ml | Absent | ＞31 days | ＞31 days | Absent |
| 12^36^ | Absent | 1.23 ng/ml | 758 U/L | Absent | Absent | 32 days | 10 days | Absent |
| 13 | 0.750ng/ml | Absent | 7420U/L | Absent | 10 days | 4 days | Normal | Absent |
| 14 | 10.500ng/ml | Absent | 11601U/L | 3050.0pg/ml | 6 days | 5 days | ＞35 days | Absent |
| 15 | 0.949ng/ml | Absent | 5346U/L | Absent | 5 days | 4 days | Normal | Absent |
| 16 | 15ng/ml | Absent | Absent | 2700.0pg/ml | 6 days | 5 days | ＞20 days | Absent |
| 17 | 6.37ng/ml | Absent | Absent | 6590.0pg/ml | 23 days | Absent | ＞13 days | Absent |

Cases 13-17 represent the five patients from our institution as reported above.

"Absent" denotes the absence of specific information in the literature. "Unknown" indicates that the literature mentions the item but lacks explicit details.

The number of days with a decrease of ≥90% in each parameter in the table was inferred based on the textual or graphical data extracted from the literature.

**Supplementary table 5: General Evolution of cTnI/T, CK/NT-proBNP in Cases of Low-Dose Group**

| **Case** | **The maximum value of cTnI before mPSL treatment** | **The maximum value of cTnT before mPSL treatment** | **The maximum value of CK before mPSL treatment** | **The maximum value of NT-proBNP before mPSL treatment** | **The number of days after initial mPSL treatment when cTnI decreases by ≥90%** | **The number of days after initial mPSL treatment when cTnT decreases by ≥90%** | **The number of days after initial mPSL treatment when CK decreases by ≥90%** | **The number of days after initial mPSL treatment when NT-proBNP decreases by ≥90%** |
| --- | --- | --- | --- | --- | --- | --- | --- | --- |
| 1^69^ | 0.44ng/ml | Absent | 335 U/L | Absent | ＞150 days | Absent | Unkonw | Absent |
| 2^70^ | 6.5 ng/ml | Absent | 1396 U/L | Absent | ＞13 days | Absent | ＞20 days | Absent |
| 3^71^ | 0.3625ng/ml | Absent | 22535.71 U/L | Absent | Unkonw | Absent | ＞10 days | Absent |
| 4^72^ | 0.87 ng/ml | Absent | 938 U/L | Absent | ＞17 days | Absent | ＞17 days | Absent |
| 5^73^ | 0.86 ng/mL | Absent | 2425 U/L | Absent | Unkonw | Absent | ＞4 days | Absent |
| 6^74^ | ＞30 ng/mL | Absent | ＞10000 U/L | Absent | ＞10 days | Absent | ＞10 days | Absent |
| 7^75^ | 10.318ng/ml | Absent | 3887U/L | Absent | ＞17 days | Absent | ＞7 days | Absent |
| 8^77^ | 2.35 ng/mL | Absent | 1658 U/L | Absent | ＞10 days | Absent | ＞30 days | Absent |
| 9^78^ | 2.796 ng/mL | Absent | 25200 U/L | Absent | ＞16 days | Absent | ＞6 days | Absent |
| 10^79^ | 1.0265 ng/mL | Absent | 510 U/L | Absent | ＞7 days | Absent | Unkonw | Absent |
| 11^80^ | 9.64 ng/ml | Absent | 4256.0 U/L | Absent | ＞15 days | Absent | ＞7 days | Absent |
| 12^81^ | 0.153 ng/ml | Absent | 1398 U/L | Absent | 0 days | Absent | ＜8 days | Absent |
| 13^82^ | Absent | 2.45ng/ml | 2940U/L | Absent | ＞7 days | ＞28 days | ＞3 days | Absent |
| 14^83^ | 0.20083 ng/ml | Absent | Normal | Absent | 7 days | Absent | 0 days | Absent |
| 15^93^ | Absent | 1.616ng/ml | ＞1400 U/L | Absent | Absent | ＞81 days | ＞81 days | Absent |
| 16^95^ | Absent | 0.988 ng/ml | 1700 U/L | 4313 pg/ml | Absent | ＞39 days | Absent | ＞15 days |
| 17^96^ | Absent | 0.031 ng/ml | 1831U/L | Normal | Absent | ＞95 days | ＞9 days | Absent |
| 18^97^ | Absent | 2.23 ng/ml | 4977 U/L | Absent | Absent | ＞49 days | ＞5 days | Absent |
| 19^98^ | Absent | 0.379 ng/ml | Absent | 474 U/L | Absent | 11 days | Absent | 1 days |
| 20^99^ | Absent | 1.128 ng/ml | 3113 U/L | Absent | Absent | 5 days | 5 days | Absent |
| 21^105^ | Absent | 1.29 ng/ml | Absent | 581.8 U/L | Absent | ＞60 days | Absent | ＞60 days |
| 22^110^ | Absent | 2.83 ng/ml | 6016 U/L | Absent | Absent | ＞40 days | ＞5 days | Absent |
| 23^113^ | Absent | 0.113 ng/ml | Absent | 350 U/L | Absent | ＞56 days | Absent | 1 days |

"Absent" denotes the absence of specific information in the literature. "Unknown" indicates that the literature mentions the item but lacks explicit details.

The number of days with a decrease of ≥90% in each parameter in the table was inferred based on the textual or graphical data extracted from the literature.

References:

1. Sessums M, Yarrarapu S, Guru PK, Sanghavi DK. Atezolizumab-induced myositis and myocarditis in a patient with metastatic urothelial carcinoma. Article. *BMJ Case Reports*. 2020;13(12)doi:10.1136/bcr-2020-236357

2. Lorente-Ros Á, Rajjoub-Al-Mahdi EA, Monteagudo Ruiz JM, et al. Checkpoint Immunotherapy-Induced Myocarditis and Encephalitis Complicated With Complete AV Block: Not All Hope Is Lost. Article. *JACC: Case Reports*. 2022;4(16):1032-1036. doi:10.1016/j.jaccas.2022.04.020

3. Khoury ZH, Hausner PF, Idzik-Starr CL, et al. Combination Nivolumab/Ipilimumab Immunotherapy For Melanoma With Subsequent Unexpected Cardiac Arrest: A Case Report and Review of Literature. *J Immunother*. Oct 2019;42(8):313-317. doi:10.1097/cji.0000000000000282

4. Katsume Y, Isawa T, Toi Y, et al. Complete atrioventricular block associated with pembrolizumab-induced acute myocarditis: The need for close cardiac monitoring. Article. *Internal Medicine*. 2018;57(21):3157-3162. doi:10.2169/internalmedicine.0255-17

5. Salido Iniesta M, López López L, Carreras Costa F, Sionis A. A different type of acute myocarditis: a case report of acute autoimmune myocarditis mediated by anti-PD-1 T lymphocyte receptor (pembrolizumab). *Eur Heart J Case Rep*. Oct 2020;4(5):1-6. doi:10.1093/ehjcr/ytaa214

6. Bae S, Vaysblat M, Ng J, Beccarino N, Makaryus J, Sarkar K. Durvalumab-Associated Myocarditis Initially Presenting With Sinus Bradycardia Progressing Into Complete Heart Block. *Cureus*. Jun 2023;15(6):e40171. doi:10.7759/cureus.40171

7. Imai R, Ono M, Nishimura N, Suzuki K, Komiyama N, Tamura T. Fulminant Myocarditis Caused by an Immune Checkpoint Inhibitor: A Case Report With Pathologic Findings. Letter. *Journal of Thoracic Oncology*. 2019;14(2):e36-e38. doi:10.1016/j.jtho.2018.10.156

8. Ganatra S, Neilan TG. Immune Checkpoint Inhibitor-Associated Myocarditis. Article. *Oncologist*. 2018;23(8):879-886. doi:10.1634/theoncologist.2018-0130

9. Chatzantonis G, Evers G, Meier C, et al. Immune Checkpoint Inhibitor-Associated Myocarditis: A Run of Bad Luck or Rather Deficient-Monitoring Protocol? Article. *JACC: Case Reports*. 2020;2(4):630-635. doi:10.1016/j.jaccas.2019.12.047

10. Deharo F, Carvelli J, Cautela J, et al. Immune Checkpoint Inhibitor-Induced Myositis/Myocarditis with Myasthenia Gravis-like Misleading Presentation: A Case Series in Intensive Care Unit. Article. *Journal of Clinical Medicine*. 2022;11(19)doi:10.3390/jcm11195611

11. Yamaguchi S, Morimoto R, Okumura T, et al. Late-Onset Fulminant Myocarditis With Immune Checkpoint Inhibitor Nivolumab. Article. *Canadian Journal of Cardiology*. 2018;34(6):812.e1-812.e3. doi:10.1016/j.cjca.2018.03.007

12. Mirabel M, Callon D, Bruneval P, et al. Late-Onset Giant Cell Myocarditis Due to Enterovirus During Treatment With Immune Checkpoint Inhibitors. Review. *JACC: CardioOncology*. 2020;2(3):511-514. doi:10.1016/j.jaccao.2020.05.022

13. Kee W, Ng KYY, Lee JJX, Tan DSW. Myasthenia Gravis and Myocarditis After Administration of Pembrolizumab in a Patient With Metastatic Non-small Cell Lung Cancer and Resected Thymoma. Article. *Clinical Lung Cancer*. 2022;23(4):e293-e295. doi:10.1016/j.cllc.2021.12.001

14. Yanase T, Moritoki Y, Kondo H, Ueyama D, Akita H, Yasui T. Myocarditis and myasthenia gravis by combined nivolumab and ipilimumab immunotherapy for renal cell carcinoma: A case report of successful management. *Urol Case Rep*. Jan 2021;34:101508. doi:10.1016/j.eucr.2020.101508

15. Bharathidasan K, Abdelnabi M, Abdelmalek J, et al. Nivolumab-induced fatal myocarditis: A case report. Article. *Clinical Case Reports*. 2023;11(5)doi:10.1002/ccr3.7306

16. Thota A, Kumar S, Pastores S. PEMBROLIZUMAB-INDUCED MYOCARDITIS WITH CONCOMITANT MYOSITIS AND HEPATITIS. Conference Abstract. *Critical Care Medicine*. 2023;51(1):371. doi:10.1097/01.ccm.0000908780.53161.d7

17. Abulnaja R. Stage 4 Non-small Cell Lung Cancer With Human Epidermal Growth Factor Receptor 2 Alterations and Myocarditis Induced by Immune Checkpoint Inhibitors: A Case Report. *Cureus*. Nov 2023;15(11):e48859. doi:10.7759/cureus.48859

18. Ederhy S, Cautela J, Ancedy Y, Escudier M, Thuny F, Cohen A. Takotsubo-Like Syndrome in Cancer Patients Treated With Immune Checkpoint Inhibitors. Article. *JACC: Cardiovascular Imaging*. 2018;11(8):1187-1190. doi:10.1016/j.jcmg.2017.11.036

19. Masood A, Mootoo A, Maghsoudlou P, et al. The threat of triple M and autoimmune overlap syndromes with immune checkpoint inhibitors - A series of case reports. Letter. *Autoimmunity Reviews*. 2023;22(3)doi:10.1016/j.autrev.2023.103269

20. Fox B, Backes F. Varying presentations of immune checkpoint inhibitor-associated myocarditis: A case report of the clinical characteristics and outcomes of three patients. Article. *Gynecologic Oncology Reports*. 2023;49doi:10.1016/j.gore.2023.101271

21. Doms J, Prior JO, Peters S, Obeid M. Tocilizumab for refractory severe immune checkpoint inhibitor-associated myocarditis. Letter. *Annals of Oncology*. 2020;31(9):1273-1275. doi:10.1016/j.annonc.2020.05.005

22. Ono R, Iwai Y, Yamazaki T, et al. Nivolumab-induced Myositis and Myocarditis with Positive Anti-titin Antibody and Anti-voltage-gated Potassium Channel Kv1.4 Antibody. Article. *Internal Medicine*. 2022;61(19):2973-2979. doi:10.2169/internalmedicine.8772-21

23. Nishikawa T, Kunimasa K, Ohta-Ogo K, et al. Sinus Node Dysfunction Co-occurring with Immune Checkpoint Inhibitor-associated Myocarditis. *Intern Med*. Jul 15 2022;61(14):2161-2165. doi:10.2169/internalmedicine.8575-21

24. Tsuruda T, Sato Y, Kajihara K, et al. Non-canonical Expression of Cardiac Troponin-T in Neuroendocrine Ethmoid Sinus Carcinoma Following Immune Checkpoint Blockade. Article. *Frontiers in Cardiovascular Medicine*. 2019;6doi:10.3389/fcvm.2019.00124

25. Toyoshima R, Uehara J, Matsuzaki Y, Yoshimura A, Kitano S, Yoshino K. Troponin-guided utilization of methylprednisolone pulse, intravenous immunoglobulin, and mycophenolate mofetil for successful control of immune checkpoint inhibitor–related myocarditis. Article in Press. *Journal of Dermatology*. 2024;doi:10.1111/1346-8138.17113

26. Cuenca JA, Hanmandlu A, Wegner R, et al. Management of respiratory failure in immune checkpoint inhibitors-induced overlap syndrome: a case series and review of the literature. Article. *BMC Anesthesiology*. 2023;23(1)doi:10.1186/s12871-023-02257-z

27. Lie G, Weickhardt A, Kearney L, et al. Nivolumab resulting in persistently elevated troponin levels despite clinical remission of myocarditis and myositis in a patient with malignant pleural mesothelioma: Case report. Article. *Translational Lung Cancer Research*. 2020;9(2):360-365. doi:10.21037/tlcr.2020.02.05

28. Long HD, Du YP, Wang LY, et al. Successful management of camrelizumab-induced immune-checkpoint-inhibitors-related myocarditis. Article in Press. *Journal of Oncology Pharmacy Practice*. 2023;doi:10.1177/10781552231216104

29. Liu Z, Fan Y, Guo J, Bian N, Chen D. Fulminant myocarditis caused by immune checkpoint inhibitor: a case report and possible treatment inspiration. Article. *ESC Heart Failure*. 2022;9(3):2020-2026. doi:10.1002/ehf2.13912

30. Wang S, Peng D, Zhu H, et al. Acetylcholine receptor binding antibody–associated myasthenia gravis, myocarditis, and rhabdomyolysis induced by tislelizumab in a patient with colon cancer: A case report and literature review. Article. *Frontiers in Oncology*. 2022;12doi:10.3389/fonc.2022.1053370

31. Liu S, Ma G, Wang H, Yu G, Chen J, Song W. Severe cardiotoxicity in 2 patients with thymoma receiving immune checkpoint inhibitor therapy: A case report. Article. *Medicine (United States)*. 2022;101(46):E31873. doi:10.1097/MD.0000000000031873

32. Wang Y, Qian M, Jin X, et al. Case Report: Temporary pacing using active fixation lead and invasive electrophysiology studies for immune checkpoint inhibitor associated reversible advanced atrioventricular block. Article. *Frontiers in Cardiovascular Medicine*. 2024;11doi:10.3389/fcvm.2024.1336609

33. Kondo H, Kirigaya J, Matsuzawa Y, Hibi K. Two Cases of Immune Checkpoint Inhibitor-Induced Myocarditis With Complete Atrioventricular Block. *Cureus*. Mar 2023;15(3):e36446. doi:10.7759/cureus.36446

34. Nguyen LS, Bretagne M, Arrondeau J, et al. Reversal of immune-checkpoint inhibitor fulminant myocarditis using personalized-dose-adjusted abatacept and ruxolitinib: Proof of concept. Article. *Journal for ImmunoTherapy of Cancer*. 2022;10(4)doi:10.1136/jitc-2022-004699

35. McDowall LM, Fernando SL, Ange N, Yun J, Chia KKM. Immune checkpoint inhibitor–mediated myocarditis and ventricular tachycardia storm. Article. *HeartRhythm Case Reports*. 2019;5(10):497-500. doi:10.1016/j.hrcr.2019.06.006

36. Iwasaki S, Hidaka H, Uojima H, et al. A case of immune checkpoint inhibitor-associated myocarditis after initiation of atezolizumab plus bevacizumab therapy for advanced hepatocellular carcinoma. Article. *Clinical Journal of Gastroenterology*. 2021;14(4):1233-1239. doi:10.1007/s12328-021-01442-2

37. Tadokoro T, Keshino E, Makiyama A, et al. Acute Lymphocytic Myocarditis With Anti-PD-1 Antibody Nivolumab. *Circ Heart Fail*. Oct 2016;9(10)doi:10.1161/circheartfailure.116.003514

38. Shalata W, Peled N, Gabizon I, Abu Saleh O, Kian W, Yakobson A. Associated Myocarditis: A Predictive Factor for Response? Article. *Case Reports in Oncology*. 2020;13(2):550-557. doi:10.1159/000507278

39. Jain V, Mohebtash M, Rodrigo ME, Ruiz G, Atkins MB, Barac A. Autoimmune Myocarditis Caused by Immune Checkpoint Inhibitors Treated with Antithymocyte Globulin. Article. *Journal of Immunotherapy*. 2018;41(7):332-335. doi:10.1097/CJI.0000000000000239

40. Huertas RM, Serrano CS, Perna C, Gómez AF, Gordoa TA. Cardiac toxicity of immune-checkpoint inhibitors: A clinical case of nivolumab-induced myocarditis and review of the evidence and new challenges. Article. *Cancer Management and Research*. 2019;11:4541-4548. doi:10.2147/CMAR.S185202

41. Al-Obaidi A, Parker NA, Choucair K, Alderson J, Deutsch JM. A Case of Acute Heart Failure Following Immunotherapy for Metastatic Lung Cancer. *Cureus*. May 13 2020;12(5):e8093. doi:10.7759/cureus.8093

42. Duarte T, Costa C, Gonçalves S, et al. A case of lymphocytic myocarditis in a patient treated with an immune checkpoint inhibitor, a recent class of chemotherapy agents. Article. *Revista Portuguesa de Cardiologia*. 2022;41(12):1047-1051. doi:10.1016/j.repc.2019.03.013

43. Padegimas A, Agarwal P, Fleitman J, et al. Case Series of Ventricular Tachycardia and Myocarditis From Programmed Cell-Death Protein-1 Inhibitor Treated With Infliximab. *JACC Clin Electrophysiol*. Aug 2019;5(8):989-992. doi:10.1016/j.jacep.2019.05.001

44. Szuchan C, Elson L, Alley E, et al. Checkpoint inhibitor-induced myocarditis and myasthenia gravis in a recurrent/metastatic thymic carcinoma patient: A case report. Article. *European Heart Journal - Case Reports*. 2020;4(3)doi:10.1093/ehjcr/ytaa051

45. Lehmann LH, Cautela J, Palaskas N, et al. Clinical Strategy for the Diagnosis and Treatment of Immune Checkpoint Inhibitor-Associated Myocarditis: A Narrative Review. Article. *JAMA Cardiology*. 2021;6(11):1329-1337. doi:10.1001/jamacardio.2021.2241

46. Gürdoğan M, Yalta K, Gürlertop Y, et al. Coexistence of Anti-PD1-Induced Immune Myocarditis and Complete Atrioventricular Block: A Case Report. Article. *American Journal of Cardiology*. 2023;207:35-38. doi:10.1016/j.amjcard.2023.08.058

47. Diamantopoulos PT, Tsatsou K, Benopoulou O, et al. Concomitant development of neurologic and cardiac immune-related adverse effects in patients treated with immune checkpoint inhibitors for melanoma. Article. *Melanoma Research*. 2020;30(5):484-491. doi:10.1097/CMR.0000000000000681

48. Tan NYL, Anavekar NS, Wiley BM. Concomitant myopericarditis and takotsubo syndrome following immune checkpoint inhibitor therapy. Article. *BMJ Case Reports*. 2020;13(9)doi:10.1136/bcr-2020-235265

49. Cham J, Ng D, Nicholson L. Durvalumab-induced myocarditis, myositis, and myasthenia gravis: a case report. Article. *Journal of Medical Case Reports*. 2021;15(1)doi:10.1186/s13256-021-02858-7

50. Leaver PJ, Jang HS, Vernon ST, Fernando SL. Immune checkpoint inhibitor-mediated myasthenia gravis with focal subclinical myocarditis progressing to symptomatic cardiac disease. *BMJ Case Rep*. May 13 2020;13(5)doi:10.1136/bcr-2019-232920

51. Wakefield C, Shultz C, Patel B, Malla M. Life-threatening immune checkpoint inhibitor-induced myocarditis and myasthenia gravis overlap syndrome treated with abatacept: A case report. Article. *BMJ Case Reports*. 2021;14(11)doi:10.1136/bcr-2021-244334

52. Inayat F, Masab M, Gupta S, Ullah W. New drugs and new toxicities: Pembrolizumab-induced myocarditis. Article. *BMJ Case Reports*. 2018;2018doi:10.1136/bcr-2017-223252

53. Valenti-Azcarate R, Esparragosa Vazquez I, Toledano Illan C, Idoate Gastearena MA, Gállego Pérez-Larraya J. Nivolumab and Ipilimumab-induced myositis and myocarditis mimicking a myasthenia gravis presentation. Article. *Neuromuscular Disorders*. 2020;30(1):67-69. doi:10.1016/j.nmd.2019.10.006

54. Delgado-Lazo V, Abdelmottaleb W, Popescu-Martinez A. Pembrolizumab-Induced Myocarditis and Pancreatitis in a Patient With Colon Cancer: A Case Report. *Cureus*. Jun 2022;14(6):e26034. doi:10.7759/cureus.26034

55. Saad R, Ghaddar A, Zeenny RM. Pembrolizumab-induced myocarditis with complete atrioventricular block and concomitant myositis in a metastatic bladder cancer patient: a case report and review of the literature. Article. *Journal of Medical Case Reports*. 2024;18(1)doi:10.1186/s13256-024-04397-3

56. Arora P, Talamo L, Dillon P, et al. Severe combined cardiac and neuromuscular toxicity from immune checkpoint blockade: An institutional case series. Article. *Cardio-Oncology*. 2020;6(1)doi:10.1186/s40959-020-00076-6

57. Liu X, Zeng Z, Cao J, et al. Sintilimab-Induced Myocarditis in a Patient with Gastric Cancer: A Case Report and Literature Review. Article. *Journal of Cardiovascular Development and Disease*. 2023;10(10)doi:10.3390/jcdd10100422

58. Tay RY, Blackley E, McLean C, et al. Successful use of equine anti-thymocyte globulin (ATGAM) for fulminant myocarditis secondary to nivolumab therapy. *Br J Cancer*. Sep 26 2017;117(7):921-924. doi:10.1038/bjc.2017.253

59. Serzan M, Rapisuwon S, Krishnan J, Chang IC, Barac A. Takotsubo Cardiomyopathy Associated With Checkpoint Inhibitor Therapy: Endomyocardial Biopsy Provides Pathological Insights to Dual Diseases. Review. *JACC: CardioOncology*. 2021;3(2):330-334. doi:10.1016/j.jaccao.2021.02.005

60. Kato S, Fukui K, Kodama S, et al. Acute Myocarditis by Immune Checkpoint Inhibitor Identified by Quantitative Pixel-Wise Analysis of Native T1 Mapping. Article. *Circulation: Cardiovascular Imaging*. 2021;14(5):E012177. doi:10.1161/CIRCIMAGING.120.012177

61. Luecke E, Ganzert C, Vielhaber S, et al. Immune Checkpoint Inhibitor-induced Fatal Myositis in a Patient With Squamous Cell Carcinoma and a History of Thymoma. Article. *Clinical Lung Cancer*. 2020;21(4):e246-e249. doi:10.1016/j.cllc.2020.01.008

62. Fazel M, Jedlowski PM. Severe Myositis, Myocarditis, and Myasthenia Gravis with Elevated Anti-Striated Muscle Antibody following Single Dose of Ipilimumab-Nivolumab Therapy in a Patient with Metastatic Melanoma. Article. *Case Reports in Immunology*. 2019;2019doi:10.1155/2019/2539493

63. Giancaterino S, Abushamat F, Duran J, Lupercio F, DeMaria A, Hsu JC. Complete heart block and subsequent sudden cardiac death from immune checkpoint inhibitor-associated myocarditis. *HeartRhythm Case Rep*. Oct 2020;6(10):761-764. doi:10.1016/j.hrcr.2020.07.015

64. Wang Q, Hu B. Successful therapy for autoimmune myocarditis with pembrolizumab treatment for nasopharyngeal carcinoma. Article. *Annals of Translational Medicine*. 2019;7(11)doi:10.21037/atm.2019.04.73

65. Jespersen MS, Fanø S, Stenør C, Møller AK. A case report of immune checkpoint inhibitor-related steroid-refractory myocarditis and myasthenia gravis-like myositis treated with abatacept and mycophenolate mofetil. Article. *European Heart Journal - Case Reports*. 2021;5(11)doi:10.1093/ehjcr/ytab342

66. Johnson DB, Balko JM, Compton ML, et al. Fulminant myocarditis with combination immune checkpoint blockade. Article. *New England Journal of Medicine*. 2016;375(18):1749-1755. doi:10.1056/NEJMoa1609214

67. Arponen O, Skyttä T. Immune checkpoint inhibitor-induced myocarditis not visible with cardiac magnetic resonance imaging but detected with PET-CT: a case report. Note. *Acta Oncologica*. 2020;59(4):490-492. doi:10.1080/0284186X.2019.1711174

68. Shalata W, Steckbeck R, Abu Salman A, et al. Perimyocarditis Associated with Immune Checkpoint Inhibitors: A Case Report and Review of the Literature. *Medicina (Kaunas)*. Jan 28 2024;60(2)doi:10.3390/medicina60020224

69. Norwood TG, Westbrook BC, Johnson DB, et al. Smoldering myocarditis following immune checkpoint blockade. *J Immunother Cancer*. Nov 21 2017;5(1):91. doi:10.1186/s40425-017-0296-4

70. Tanabe J, Watanabe N, Endo A, Nagami T, Inagaki S, Tanabe K. Asymptomatic immune checkpoint inhibitor-associated myocarditis. Article. *Internal Medicine*. 2021;60(4):569-573. doi:10.2169/internalmedicine.5412-20

71. Wang C, Zhong B, He J, Liao X. Immune checkpoint inhibitor sintilimab-induced lethal myocarditis overlapping with myasthenia gravis in thymoma patient: A case report. Article. *Medicine (United States)*. 2023;102(15):E33550. doi:10.1097/MD.0000000000033550

72. Ye Y, Li Y, Zhang S, Han G. Teriprizumab-induced myocarditis in a patient with cholangiocarcinoma: a case report. Article. *Journal of International Medical Research*. 2022;50(10)doi:10.1177/03000605221133259

73. Ji H, Wen Z, Liu B, Chen H, Lin Q, Chen Z. Sintilimab induced ICIAM in the treatment of advanced HCC: A case report and analysis of research progress. Article. *Frontiers in Immunology*. 2022;13doi:10.3389/fimmu.2022.995121

74. Saibil SD, Bonilla L, Majeed H, et al. Fatal myocarditis and rhabdomyositis in a patient with stage IV melanoma treated with combined ipilimumab and nivolumab. *Curr Oncol*. Jun 2019;26(3):e418-e421. doi:10.3747/co.26.4381

75. Matsui H, Kawai T, Sato Y, et al. A fatal case of myocarditis following myositis induced by pembrolizumab treatment for metastatic upper urinary tract urothelial carcinoma. Article. *International Heart Journal*. 2020;61(5):1070-1074. doi:10.1536/ihj.20-162

76. Hyun JW, Kim GS, Kim SH, et al. Fatal Simultaneous Multi-organ Failure Following Pembrolizumab Treatment for Refractory Thymoma. *Clin Lung Cancer*. Mar 2020;21(2):e74-e77. doi:10.1016/j.cllc.2019.10.008

77. Yin B, Xiao J, Wang X, et al. Myocarditis and myositis/myasthenia gravis overlap syndrome induced by immune checkpoint inhibitor followed by esophageal hiatal hernia: A case report and review of the literature. Article. *Frontiers in Medicine*. 2022;9doi:10.3389/fmed.2022.950801

78. Luo YB, Tang W, Zeng Q, et al. Case Report: The Neuromusclar Triad of Immune Checkpoint Inhibitors: A Case Report of Myositis, Myocarditis, and Myasthenia Gravis Overlap Following Toripalimab Treatment. Article. *Frontiers in Cardiovascular Medicine*. 2021;8doi:10.3389/fcvm.2021.714460

79. Su L, Liu C, Wu W, Cui Y, Wu M, Chen H. Successful Therapy for Myocarditis Concomitant With Complete Heart Block After Pembrolizumab Treatment for Head and Neck Squamous Cell Carcinoma: A Case Report With Literature Review. Article. *Frontiers in Cardiovascular Medicine*. 2022;9doi:10.3389/fcvm.2022.898756

80. Xie X, Wang F, Qin Y, et al. Case Report: Fatal Multiorgan Failure and Heterochronous Pneumonitis Following Pembrolizumab Treatment in a Patient With Large-Cell Neuroendocrine Carcinoma of Lung. Article. *Frontiers in Pharmacology*. 2020;11doi:10.3389/fphar.2020.569466

81. Yin N, Liu X, Ye X, Song W, Lu J, Chen X. PD-1 inhibitor therapy causes multisystem immune adverse reactions: a case report and literature review. Article. *Frontiers in Oncology*. 2022;12doi:10.3389/fonc.2022.961266

82. Ang E, Mweempwa A, Heron C, et al. Cardiac Troponin i and T in Checkpoint Inhibitor-associated Myositis and Myocarditis. Article. *Journal of Immunotherapy*. 2021;44(4):162-163. doi:10.1097/CJI.0000000000000356

83. Zhou B, Li M, Chen T, She J. Case Report: Acute Myocarditis Due to PD-L1 Inhibitor Durvalumab Monotherapy in a Patient With Lung Squamous Cell Carcinoma. *Front Med (Lausanne)*. 2022;9:866068. doi:10.3389/fmed.2022.866068

84. Bi H, Ren D, Wang Q, Ding X, Wang H. Immune checkpoint inhibitor-induced myocarditis in lung cancer patients: a case report of sintilimab-induced myocarditis and a review of the literature. Article. *Annals of palliative medicine*. 2021;10(1):793-802. doi:10.21037/apm-20-2449

85. Bai J, Li D, Yang P, et al. Camrelizumab-Related Myocarditis and Myositis With Myasthenia Gravis: A Case Report and Literature Review. Article. *Frontiers in Oncology*. 2021;11doi:10.3389/fonc.2021.778185

86. Zhang B, Gyawali L, Liu Z, Du H, Yin Y. Camrelizumab-Related Lethal Arrhythmias and Myasthenic Crisis in a Patient with Metastatic Thymoma. Article. *Case Reports in Cardiology*. 2022;2022doi:10.1155/2022/4042909

87. Hernández AP, Clemente MB, García DE, et al. Checkpoint inhibitor-induced fulminant myocarditis, complete atrioventricular block and myasthenia gravis-a case report. Article. *Cardiovascular Diagnosis and Therapy*. 2021;11(4):1013-1019. doi:10.21037/cdt-21-147

88. Sato T, Nakamori S, Watanabe S, et al. Monitoring of the Evolution of Immune Checkpoint Inhibitor Myocarditis With Cardiovascular Magnetic Resonance. Article. *Circulation: Cardiovascular Imaging*. 2020;13(11):E010633. doi:10.1161/CIRCIMAGING.120.010633

89. Chen Y, Chen Y, Xie J, Liu D, Hong X. Multisystem immune-related adverse events due to toripalimab: Two cases-based review. Article. *Frontiers in Cardiovascular Medicine*. 2022;9doi:10.3389/fcvm.2022.1036603

90. Aghel N, Gustafson D, Di Meo A, et al. Recurrent Myocarditis Induced by Immune-Checkpoint Inhibitor Treatment Is Accompanied by Persistent Inflammatory Markers Despite Immunosuppressive Treatment. *JCO Precis Oncol*. Mar 2021;5doi:10.1200/po.20.00370

91. Giblin GT, Dennehy C, Featherstone H, et al. Subclinical Myocarditis After Combination Immune Checkpoint Inhibitor Therapy. Article. *Circulation: Heart Failure*. 2021;14(2):E007524. doi:10.1161/CIRCHEARTFAILURE.120.007524

92. Ida M, Nakamori S, Yamamoto S, et al. Subtle-but-smouldering myocardial injury after immune checkpoint inhibitor treatment accompanied by amyloid deposits. Article. *ESC Heart Failure*. 2022;9(3):2027-2031. doi:10.1002/ehf2.13915

93. Salem JE, Allenbach Y, Vozy A, et al. Abatacept for Severe Immune Checkpoint Inhibitor-Associated Myocarditis. *N Engl J Med*. Jun 13 2019;380(24):2377-2379. doi:10.1056/NEJMc1901677

94. Hardy T, Yin M, Chavez JA, et al. Acute fatal myocarditis after a single dose of anti-PD-1 immunotherapy, autopsy findings: a case report. Article. *Cardiovascular Pathology*. 2020;46doi:10.1016/j.carpath.2020.107202

95. Yang Y, Xu L, Wang D, et al. Anti-PD-1 and regorafenib induce severe multisystem adverse events in microsatellite stability metastatic colorectal cancer: a case report. *Immunotherapy*. Nov 2021;13(16):1317-1323. doi:10.2217/imt-2020-0327

96. Shindo A, Yamasaki M, Uchino K, Yamasaki M. Asymptomatic Myocarditis with Mild Cardiac Marker Elevation Following Nivolumab-Induced Myositis. Article. *International Heart Journal*. 2022;63(1):180-183. doi:10.1536/ihj.21-653

97. Zheng S, Zhang H, Hu B, Zhou J, Wen L, Li M. A case of acute myocarditis induced by PD-1 inhibitor (sintilimab) in the treatment of large cell neuroendocrine carcinoma. *Heliyon*. Jun 2023;9(6):e16874. doi:10.1016/j.heliyon.2023.e16874

98. Hu Y, Liu C, Jin S, et al. A case of subclinical immune checkpoint inhibitor-associated myocarditis in non-small cell lung cancer. Article. *BMC Pulmonary Medicine*. 2023;23(1)doi:10.1186/s12890-023-02417-4

99. Ruperti-Repilado FJ, Van Der Stouwe JG, Haaf P, et al. Case report of elevation of high-sensitivity cardiac troponin T in the absence of cardiac involvement in immune checkpoint inhibitor-associated myositis. Article. *European Heart Journal - Case Reports*. 2022;6(9)doi:10.1093/ehjcr/ytac353

100. Nishiyama K, Morikawa K, Shinozaki Y, et al. Case report: Electrocardiographic changes in pembrolizumab-induced fatal myocarditis. Article. *Frontiers in Immunology*. 2023;14doi:10.3389/fimmu.2023.1078838

101. Xing Q, Zhang Z, Zhu B, et al. Case Report: Treatment for steroid-refractory immune-related myocarditis with tofacitinib. Article. *Frontiers in Immunology*. 2022;13doi:10.3389/fimmu.2022.944013

102. Zomborska E, Kasperova S, Slopovsky J, et al. Fatal myocarditis after the first dose of nivolumab. Article. *Klinicka Onkologie*. 2022;35(6):486-492. doi:10.48095/ccko2022486

103. Nelke C, Pawlitzki M, Kerkhoff R, et al. Immune Checkpoint Inhibition-Related Myasthenia-Myositis-Myocarditis Responsive to Complement Blockade. Article. *Neurology(R) neuroimmunology & neuroinflammation*. 2024;11(1)doi:10.1212/NXI.0000000000200177

104. Gao L, Li X, Guo Z, Tang L, Peng J, Liu B. Immune checkpoint inhibitor-induced myocarditis with myasthenia gravis overlap syndrome: A case report and literature review. Article. *Medicine (United States)*. 2022;101(49):E32240. doi:10.1097/MD.0000000000032240

105. Liang S, Yang J, Lin Y, et al. Immune Myocarditis Overlapping With Myasthenia Gravis Due to Anti-PD-1 Treatment for a Chordoma Patient: A Case Report and Literature Review. Article. *Frontiers in Immunology*. 2021;12doi:10.3389/fimmu.2021.682262

106. Zhang C, Qin S, Zuo Z. Immune-related myocarditis in two patients receiving camrelizumab therapy and document analysis. Article. *Journal of Oncology Pharmacy Practice*. 2022;28(6):1350-1356. doi:10.1177/10781552211027339

107. Delombaerde D, Vervloet D, Berwouts D, et al. Ipilimumab- and nivolumab-induced myocarditis in a patient with metastatic cholangiocarcinoma: a case report. Article. *Journal of Medical Case Reports*. 2022;16(1)doi:10.1186/s13256-022-03487-4

108. Baldessari C, Pugliese G, Venturelli M, et al. Myocarditis and diaphragmatic rhabdomyolysis with respiratory failure in a patient with metastatic melanoma treated with Nivolumab. Article. *Journal of Oncology Pharmacy Practice*. 2022;28(3):750-753. doi:10.1177/10781552211067424

109. Chen Y, Jia Y, Liu Q, et al. Myocarditis related to immune checkpoint inhibitors treatment: two case reports and literature review. Article. *Annals of palliative medicine*. 2021;10(7):8512-8517. doi:10.21037/apm-20-2620

110. Cao J, Li Q, Zhi X, et al. Pembrolizumab-induced autoimmune Stevens-Johnson syndrome/toxic epidermal necrolysis with myositis and myocarditis in a patient with esophagogastric junction carcinoma: A case report. Article. *Translational Cancer Research*. 2021;10(8):3870-3876. doi:10.21037/tcr-21-470

111. Schiopu SRI, Käsmann L, Schönermarck U, et al. Pembrolizumab-induced myocarditis in a patient with malignant mesothelioma: Plasma exchange as a successful emerging therapy-case report. Article. *Translational Lung Cancer Research*. 2021;10(2):1039-1046. doi:10.21037/tlcr-20-1095

112. Yang Y, Wu Q, Chen L, Qian K, Xu X. Severe immune-related hepatitis and myocarditis caused by PD-1 inhibitors in the treatment of triple-negative breast cancer: a case report. Article. *Annals of Translational Medicine*. 2022;10(7)doi:10.21037/atm-22-1284

113. Liu Y, Jiang L. Tofacitinib for treatment in immune-mediated myocarditis: The first reported cases. *J Oncol Pharm Pract*. Aug 11 2020:1078155220947141. doi:10.1177/1078155220947141

114. Zhao LZ, Liu G, Li QF, Chen G, Jin GW. A case of carrelizumab-associated immune myocarditis. *Asian J Surg*. Jan 2022;45(1):496-497. doi:10.1016/j.asjsur.2021.08.067

115. Elder CT, Davis EC, Jaipal S, Wight CE. Immune-checkpoint inhibitor toxicity during a pandemic: Overcoming patient fears to provide care. A case report. Article. *Journal of Oncology Pharmacy Practice*. 2021;27(8):2035-2040. doi:10.1177/10781552211012782

116. Moriyama S, Fukata M, Tatsumoto R, Kono M. Refractory constrictive pericarditis caused by an immune checkpoint inhibitor properly managed with infliximab: a case report. Article. *European Heart Journal - Case Reports*. 2021;5(1)doi:10.1093/ehjcr/ytab002

117. Prevel R, Colin G, Calès V, Renault PA, Mazieres J. Third degree atrio-ventricular blockade during a myocarditis occurring under anti-PD1 : Case report and literature review. Article. *Revue de Medecine Interne*. 2020;41(4):284-288. doi:10.1016/j.revmed.2019.12.023
